# Perturbation of *Pseudomonas aeruginosa* peptidoglycan recycling by anti-folates and design of a dual-action inhibitor

**DOI:** 10.1128/mbio.02984-24

**Published:** 2025-01-29

**Authors:** Luke N. Yaeger, David Sychantha, Princeton Luong, Shahrokh Shekarriz, Océane Goncalves, Annamaria Dobrin, Michael R. Ranieri, Ryan P. Lamers, Hanjeong Harvey, George C. diCenzo, Michael Surette, Jean-Phiippe Côté, Jakob Magolan, Lori L. Burrows

**Affiliations:** 1 Department of Biochemistry and Biomedical Sciences, McMaster University, Hamilton, Ontario, Canada; 2 Michael G. DeGroote Institute for Infectious Disease Research, McMaster University, Hamilton, Ontario, Canada; 3 Département de Biologie, Université de Sherbrooke 7321 , Sherbrooke, Québec, Canada; 4 Department of Biology, Queen’s University, Kingston, Ontario, Canada; Emory University, Atlanta, Georgia, USA; Geisel School of Medicine at Dartmouth, Hanover, New Hampshire, USA

**Keywords:** *Pseudomonas aeruginosa*, *Escherichia coli*, cell wall, antibiotic resistance, antibiotic combinations, folate, purines, synergy, chemical biology, beta-lactamase, metallo-beta-lactamase

## Abstract

Peptidoglycan (PG) is an important bacterial macromolecule that confers cell shape and structural integrity, and is a key antibiotic target. Its synthesis and turnover are carefully coordinated with other cellular processes and pathways. Despite established connections between the biosynthesis of PG and the outer membrane, or PG and DNA replication, links between PG and folate metabolism remain comparatively unexplored. Folate is an essential cofactor for bacterial growth and is required for the synthesis of many important metabolites. Here we show that inhibition of folate synthesis in the important Gram-negative pathogen *Pseudomonas aeruginosa* has downstream effects on PG metabolism and integrity that can manifest as the formation of a subpopulation of round cells that can undergo explosive lysis. Folate inhibitors potentiated β-lactams by perturbation of PG recycling, reducing expression of the AmpC β-lactamase. Supporting this mechanism, folate inhibitors also synergized with fosfomycin, an inhibitor of MurA, the first committed step in PG synthesis that can be bypassed by PG recycling. These insights led to the design of a dual-active inhibitor that overcomes NDM-1 metallo-β lactamase-mediated meropenem resistance and synergizes with the folate inhibitor, trimethoprim. We show that folate and PG metabolism are intimately connected, and targeting this connection can overcome antibiotic resistance in Gram-negative pathogens.

**IMPORTANCE:**

To combat the alarming global increase in superbugs amid the simultaneous scarcity of new drugs, we can create synergistic combinations of currently available antibiotics or chimeric molecules with dual activities, to minimize resistance. Here we show that older anti-folate drugs synergize with specific cell wall biosynthesis inhibitors to kill the priority pathogen, *Pseudomonas aeruginosa*. Anti-folate drugs caused a dose-dependent loss of rod cell shape followed by explosive lysis, and synergized with β-lactams that target D,D-carboxypeptidases required to tailor the cell wall. Anti-folates impaired cell wall recycling and subsequent downstream expression of the chromosomally encoded β-lactamase, AmpC, which normally destroys β-lactam antibiotics. Building on the anti-folate-like scaffold of a metallo-β-lactamase inhibitor, we created a new molecule, MLLB-2201, that potentiates β-lactams and anti-folates and restores meropenem activity against metallo-β-lactamase-expressing *Escherichia coli*. These strategies are useful ways to tackle the ongoing rise in dangerous bacterial pathogens.

## INTRODUCTION

High levels of antibiotic resistance in the opportunistic and nosocomial pathogen *Pseudomonas aeruginosa* can limit treatment options (1). The use of combination therapy can restore the efficacy of current antibiotics against resistant strains (2). In a common example, β-lactam and β-lactamase inhibitor combinations extend the spectrum of cell-wall targeting β-lactams to strains that produce antibiotic-degrading enzymes (3). Some combinations go beyond unidirectional potentiation, achieving drug-drug synergy through their mutual potentiation. For example, the antibiotics trimethoprim (TMP) and sulfamethoxazole (SUL) inhibit separate steps in folate biosynthesis, and together, the two drugs are more potent than the single agents (4).

TMP and SUL block the production of tetrahydrofolate (THF), an essential cofactor in one-carbon metabolism (https://www.genome.jp/pathway/pae00790) (5). SUL inhibits dihydropteroate synthase (FolP, EC 2.5.1.15) by mimicking its substrate para-aminobenzoic acid (PABA). FolP is two steps upstream of the TMP target, dihydrofolate reductase (FolA, also called DHFRII, EC 1.5.1.3), which catalyzes the formation of THF from dihydrofolate (DHF). THF and its derivatives act as cofactors and one-carbon donors to generate key metabolites including purines, methionine, S-adenosyl methionine, thymidylate, glycine, and serine (6). The large number of THF-dependent metabolites suggests that treatment with folate inhibitors can have far-reaching effects on cell physiology. The best-studied effect of folate inhibition is a decrease in pools of thymidylate, a metabolite required for DNA synthesis (7). By inhibiting DNA synthesis, TMP treatment can induce the SOS response, inhibiting the assembly of the cell division machinery to prevent premature completion of the cell cycle when chromosomal replication is delayed (8).

Peptidoglycan (PG) metabolism can be broadly divided into subunit synthesis, assembly, and turnover/recycling (9–11). It is an essential process that draws upon many different metabolite pools and is carefully coordinated with broader cell physiology. Here we use bioinformatics, microscopy, and chemical genetics to characterize vulnerable connections between folate and PG metabolism, showing that anti-folates specifically impact PG turnover/recycling. We leverage our findings to design a dual inhibitor that synergizes with anti-folates and overcomes Gram-negative resistance to the PG synthesis inhibitor, meropenem.

## RESULTS

### Connections between folate and peptidoglycan pathways in *P. aeruginosa*

**Fig 1:**
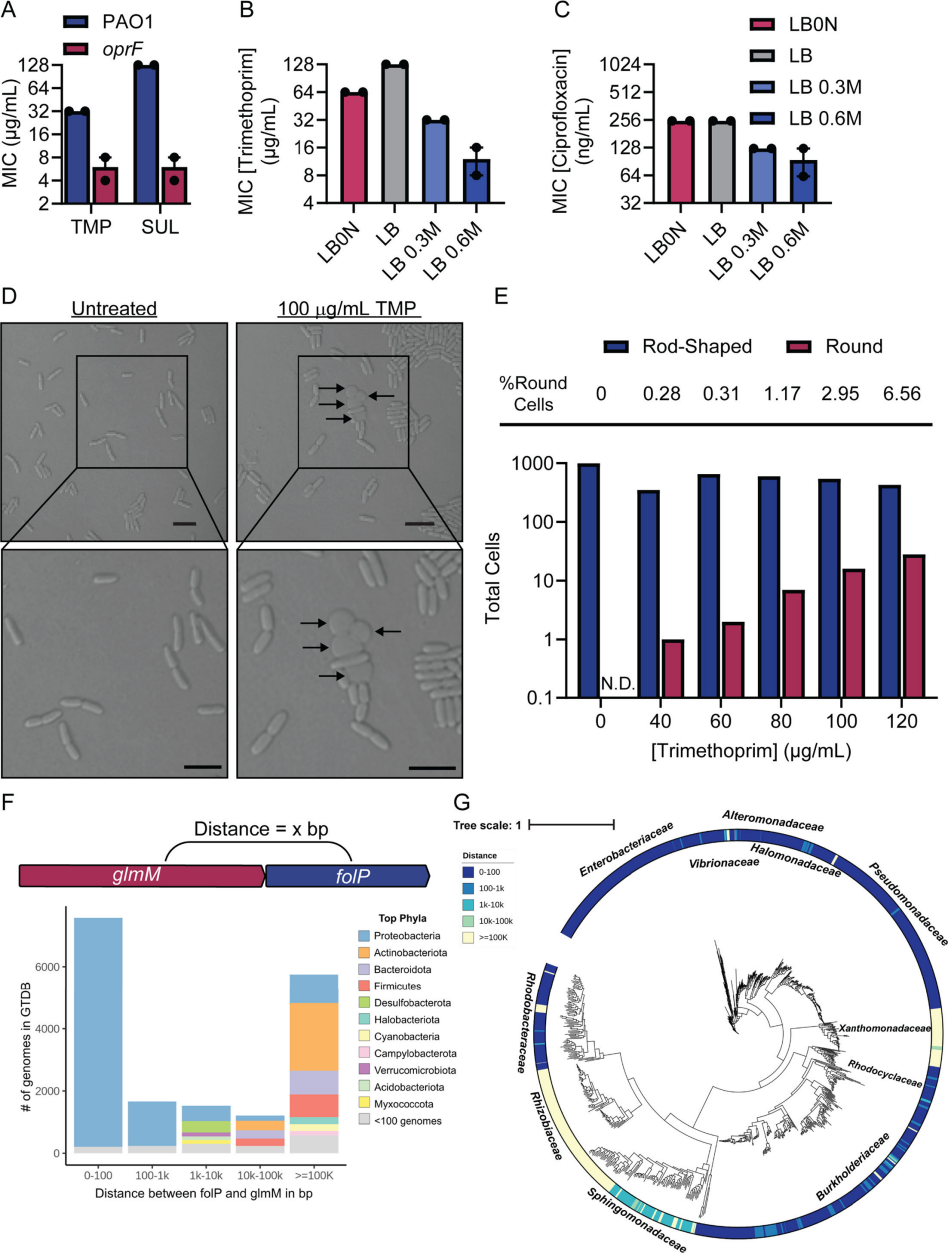
Folate inhibition perturbs cell envelope integrity. (**A**) MICs of TMP or SUL for PAO1 and the *oprF* mutant. MICs of (B) TMP or (C) CIP for PAO1 grown in LB with different NaCl concentrations (LB0N = LB with no NaCl). MICs from two biological replicates are plotted. The data points for each biological replicate are averaged from three technical replicates. (**D**) PAO1 cells treated with TMP. Scale bar = 5 µm. The arrows point to round cells. Close-ups of each image are shown below the original image. Sample preparation and imaging were performed in biological duplicates and representative micrographs are shown. (**E**) Quantification of round cell formation across a range of TMP concentrations. At least 350 cells per TMP concentration from two biological replicates with three fields of view each were counted. (**F**) Distance in base pairs between *glmM* and *folP* across diverse bacterial genomes. (**G**) Unrooted phylogenetic tree overlayed with the distance between *folP* and *glmM* mapped across representative genomes of the phylum *Proteobacteria*. Major families of the phylum *Proteobacteria* are labeled next to the corresponding genomes. The scale bar represents the average number of amino acid substitutions per site. The distance between *folP* and *glmM* is represented by a color scale, where dark blue and light yellow indicate smaller or larger intergenic distances, respectively.

We previously reported that a *P. aeruginosa oprF* mutant was hypersensitive to the anti-folate TMP, with a 16-fold decrease in its minimal inhibitory concentration (MIC) in low nutrient media compared to the parent strain (12). In contrast, susceptibility of *oprF* to antibiotics of other classes was similar to wild type. The *oprF* mutant was also hypersensitive to SUL (Fig. 1A), confirming its general sensitivity to folate inhibition. OprF is an outer membrane porin with a PG-binding domain that anchors the outer membrane to the cell wall, leading us to consider possible connections between folate and PG pathways (13). We next tested whether TMP might compromise cell envelope integrity by exposing cells to hyper- or hypo-osmotic stresses, which can impact the susceptibility of bacteria to antibiotics that impact cell wall biosynthesis (14). Increasing the NaCl concentration reduced the MIC of TMP by ~8× (Fig. 1B). This change was specific to folate inhibition, as the MIC of another antibiotic that targets DNA biosynthesis, ciprofloxacin (CIP; a DNA gyrase inhibitor), was minimally affected by high NaCl (Fig. 1C). We examined the cell morphology of TMP-treated *P. aeruginosa* by light microscopy and saw that a subset of cells lost their rod shape, reminiscent of PG-less L-forms (15) (Fig. 1D). The proportion of round cells increased with the concentration of TMP (Fig. 1E), and we saw that a subset of these round cells could undergo explosive cell lysis (Video S1). Therefore, folate inhibition resulted in phenotypes resembling those resulting from perturbation of PG metabolism.

While exploring potential folate-PG connections, we identified syntenies between multiple folate and PG genes that could indicate potential coevolution (16) (Fig. S1a). For example, PA5227 encodes a homolog of ZapA involved in cell division, while the adjacent gene PA5228 encodes a homolog of Fau, which produces the folate derivative 5,10-methenyltetrahydrofolate. PA3110, upstream of *folC* (PA3111), encodes a homolog of DedD involved in cell division, and PA2963, adjacent to *pabC* (PA2964) whose product catalyzes the production of PABA, encodes a homolog of the lytic transglycosylase, MltG, involved in PG turnover. *folP,* encoding the target of SUL, is contiguous with *glmM*, encoding a phosphoglucosamine mutase that feeds the key metabolite glucosamine 1-phosphate into PG synthesis. We measured the proximity of these two genes across >40,000 representative prokaryotic genomes. Synteny between *folP* and *glmM* was mainly constrained to the phylum Proteobacteria (Pseudomonadota), with some exceptions (Fig. 1F). Plotting the distance between *folP* and *glmM* on a tree of 938 complete proteobacterial genomes showed that the synteny was generally conserved for all but the *Xanthomonadaceae*, *Sphingomonadaceae*, and *Rhizobiaceae* (Fig. 1G). This finding expands upon a previous report that *Xanthomonas campestris* is distinct among species of the class *Gammaproteobacteria* in that *glmM* and *folP* are encoded separately (17). Some *Rhizobiaceae* species lack a *folP* homolog and presumably import folate, like some lactobacilli (18, 19). This analysis supported a relationship between folate and PG genes.

Besides genetic syntenies, we noted striking structural similarities between a subset of folate and PG enzymes that could also support evolutionary connections. FolC, the folylpolyglutamate synthase, and MurC-F both belong to the Mur ligase family (Fig. S1b) (20). MurA, the first committed step in PG synthesis, is structurally related to AroA involved in the biosynthesis of the folate precursor chorismate, and they use the same reaction mechanism for the addition of enolpyruvate (21). PabC is structurally homologous to a D-amino acid aminotransferase (22, 23) that produces D-Glu and D-Ala for the addition of the PG stem peptide. Finally, *P. aeruginosa* Cpg2 is a periplasmic carboxypeptidase that cleaves folate to produce glutamyl and pteroate groups (24); its structure resembles that of DapE, which catalyzes the production of the mDAP component of the PG stem peptide (25). Together, the syntenies and structural similarities of biosynthetic components for PG and folate support the potential co-evolution and coordination of these crucial metabolic pathways.

### A chemical-genetic screen uncovers specific interactions of PG inhibitors with TMP

**Fig 2:**
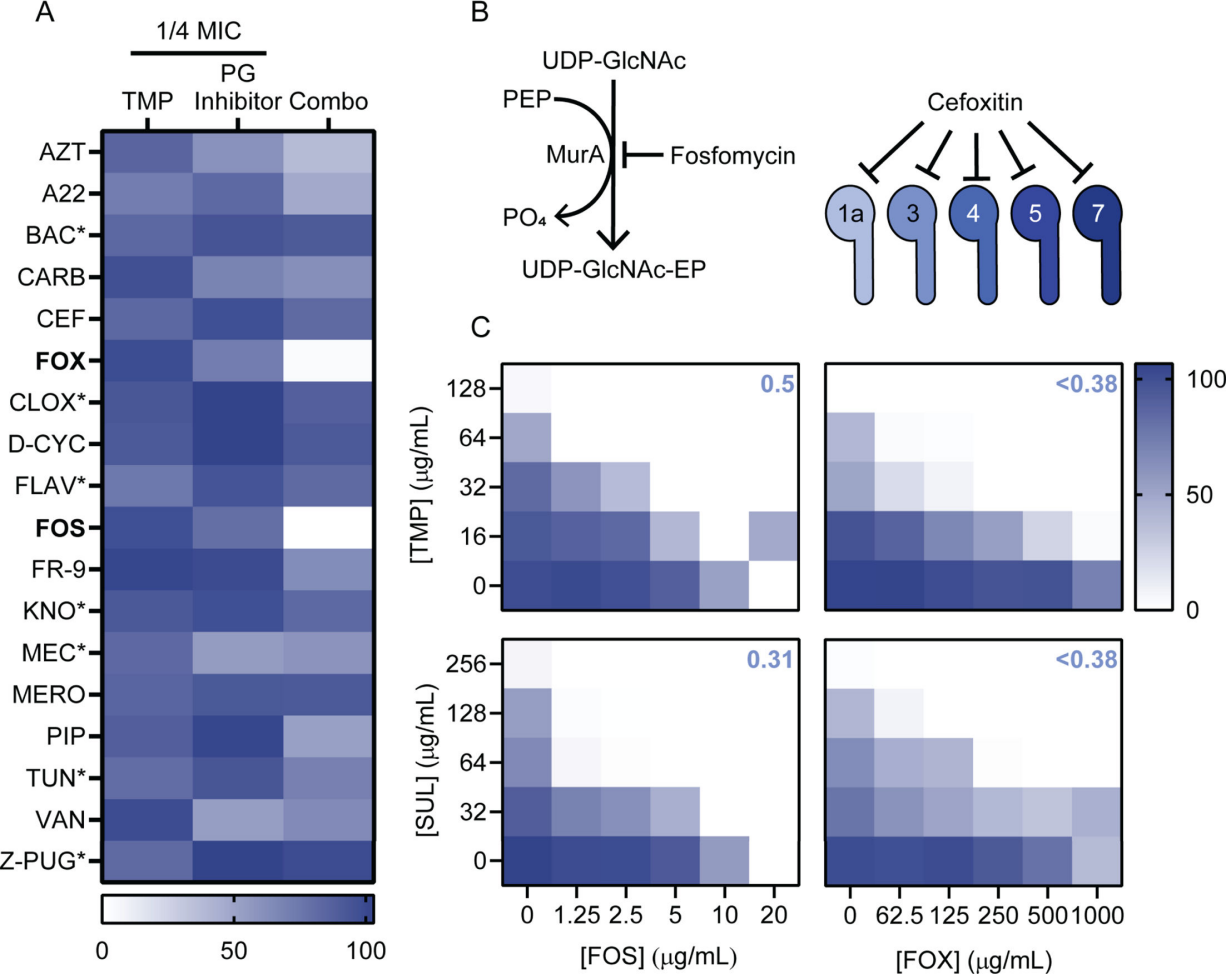
A chemical interaction screen reveals specific anti-folate/PG inhibitor potentiation. (**A**) The heatmap in (A) summarizes the data from 8 × 8 checkerboard assays with TMP and the antibiotics listed to the left of the heatmap. AZT = aztreonam, A22 = A22, BAC = bacitracin, CARB = carbenicillin, CEF = cefixime, FOX = cefoxitin, CLOX = cloxacillin, D-CYC = D-cycloserine, FLAV = flavomycin, FOS = fosfomycin, FR-9 = FR-900098, KNO = kanosamine, MEC = mecillinam, MERO = meropenem, PIP = piperacillin, TUN = tunicamycin, VAN = vancomycin, Z-PUG = Z-PUGNAc. Asterisks indicate that MIC was above the highest concentration tested. Bold font indicates inhibitors that demonstrated synergy. Growth is shown as a percent of the vehicle control. The left column shows growth with ¼ MIC TMP alone; the middle column, growth with ¼ MIC of the PG inhibitor; and the right column, with ¼ MIC of both TMP and the PG inhibitor. (**B**) Left: the reaction catalyzed by MurA, which is inhibited by fosfomycin. PEP = phosphoenolpyruvate, PO_4_ = phosphate, EP = enolpyruvate. Right: PBPs inhibited by cefoxitin, according to Ropy et al*.* (27) (C) Heatmaps from 8 × 8 checkerboards condensed to 5 × 6 checkerboards. Growth is shown as a percent of the vehicle control. Checkerboard assays were repeated in biological triplicates and representative heatmaps from one replicate are shown. FICIs are shown in the top right corner.

To probe possible mechanisms of anti-folate-induced cell wall perturbation, we first tested a collection of small molecule inhibitors that target different steps of PG metabolism for chemical-chemical interactions with TMP (Fig. 2A). Notably, fosfomycin (FOS) and cefoxitin (FOX), which target MurA and penicillin-binding proteins (PBPs) (Fig. 2B), respectively, potentiated TMP (Fig. 2C). This is a general feature of folate inhibition, as SUL also potentiated FOS and FOX (Fig. 2C). However, FOS and FOX failed to synergize with ciprofloxacin, suggesting that the synergistic effects are not related to inhibition of DNA synthesis (Fig. S2a). Overexpression of FolA, the target of TMP, increased the concentration required for potentiation (Fig. S2b), suggesting that the established mechanism of action drives the interaction. While anti-folates might synergize with FOS/FOX due to increased cell permeability, we do not believe this to be the case, because (i) we did not see potentiation of TMP/SUL or FOS by the permeabilizer polymyxin B (Fig. S2C and D); (ii) TMP failed to interact with most other cell wall-targeting antibiotics (Fig. 2A), supporting the concept that there are specific mechanisms of potentiation, and (iii) TMP potentiation was lost in a *glpT* mutant that prevents FOS from crossing the inner membrane to inhibit MurA (26) (Fig. S2e).

### TMP potentiates FOX by suppressing the AmpR/AmpC response

**Fig 3:**
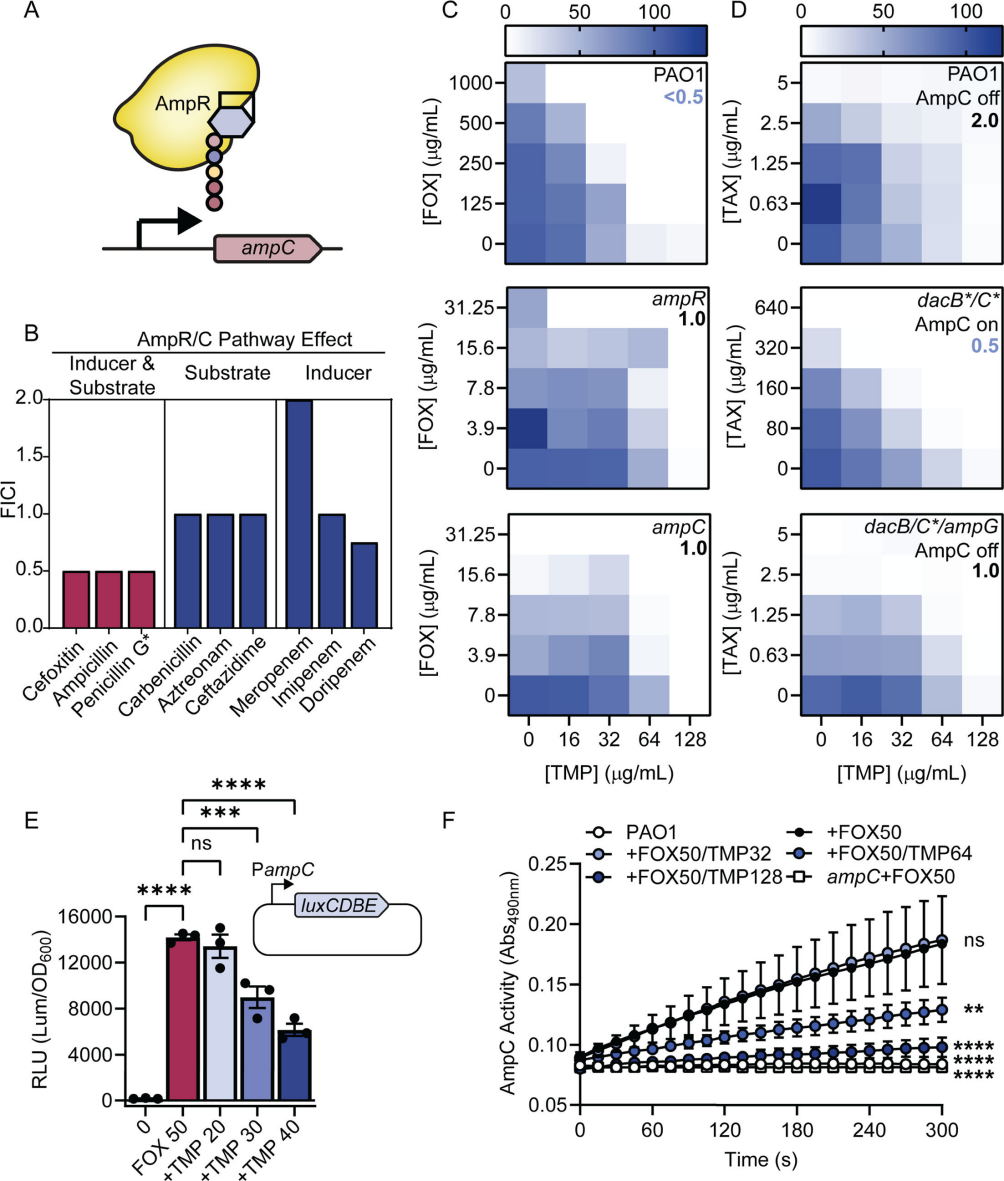
TMP potentiates β-lactams by impairing *ampC* expression. (**A**) Schematic showing activation of *ampC* transcription by AmpR bound to anhMurNAc-pentapeptide. (**B**) Summary of the fractional inhibitory concentrations (FICI) from 8 × 8 checkerboard assays of TMP and antibiotics. An asterisk indicates that an MIC was not reached for that antibiotic alone. The mean FICI was calculated from two biological replicates of 8 × 8 checkerboard assays. (**C**) Heatmaps showing 5 × 5 checkerboards that condense data from 8 × 8 checkerboards. Blue indicates growth as a percent of the vehicle control. TMP concentrations are on the bottom and FOX concentrations are on the left. Note that the FOX concentration range is lower for the susceptible *ampC* and *ampR* mutants (middle and bottom). Checkerboards were performed in biological duplicate, and heatmaps were shown for a representative replicate. (**D**) Heatmaps showing 5 × 5 checkerboards condensed from 8 × 8 checkerboards. The TAX concentration range is higher for the resistant dacBC** mutant. Checkerboards were performed in biological duplicate, and heatmaps were shown for a representative replicate. (**E**) *ampC* promoter-driven relative luminescence (RLU, calculated as arbitrary luminescence units divided by the matched OD_600_ growth value) across the different treatment conditions. A schematic showing the *ampC* promoter upstream of the *luxCDBE* genes is above the graph. “0” = no antibiotic added, FOX50 = 50 µg/mL cefoxitin, +TMP 20/30/40 = 20, 30, or 40 µg/mL TMP added in addition to 50 µg/mL FOX. The bars represent the mean RLU, individual data points are shown as black circles, and error bars are the standard error of the mean. Technical triplicates were performed for two biological replicates, and a representative replicate is shown. A one-way ANOVA followed by Dunnett’s multiple comparisons test was performed to compare all the data to the FOX50 condition. NS = not significant, *** = *P* < 0.001, **** = *P* < 0.0001. (**F**) Nitrocefin hydrolysis (measured as absorbance at 490 nm) over time. The symbols show the mean of technical duplicates, the error bars show the standard error of the mean. The circles show the wild-type strain treated as in the legend. The squares indicate the negative control *ampC* treated with cefoxitin. Experiments were performed in technical and biological duplicates, and data from a representative biological replicate are shown.

FOX, like other β-lactams, inhibits multiple PBPs (27). We next sought to understand why TMP specifically potentiated FOX but not the other β-lactams in our panel (Fig. 2A). *P. aeruginosa* is typically considered resistant to FOX, which inhibits PBPs 4 (DacB) and 5 (DacC). These PBPs cleave the terminal D-Ala residue from pentapeptide stems to limit the extent of cell wall cross-linking. Inhibiting these PBPs increases the pool of GlcNAc-anhMurNAc-pentapeptides returned to the cytoplasm by the transporter AmpG. The recycling intermediate AnhMurNAc-pentapeptide binds the regulator AmpR to induce the expression of AmpC, a β-lactamase that degrades FOX, rendering *P. aeruginosa* insensitive (28) (Fig. 3A). Of the β-lactams we tested, only FOX is both an inducer and substrate of AmpC, suggesting that anti-folates might block activation of the AmpR/AmpC pathway, preventing the bacteria from initiating their normal defensive response to β-lactam treatment.

To test this idea, we used three types of β-lactams: those that are both inducers of AmpC expression and its substrates (such as penicillin), those that do not induce AmpC but are substrates (e.g., carbenicillin), and those that induce AmpC expression but are non-substrates (e.g., meropenem). Figure 3B shows the fractional inhibitory concentrations (FICI) with each type of β-lactam in combination with TMP. An FICI of 0.5 or less is considered synergistic (29). Supporting our hypothesis, only those β-lactams that are both inducers and substrates of AmpC synergized with TMP. If AmpC and AmpR were required for the TMP-FOX interaction, then TMP should not further reduce the FOX MIC of *ampR* or *ampC* mutants. Indeed, TMP and FOX failed to synergize in those backgrounds (Fig. 3C).

Genetic inactivation of PBPs 4 and 5—by mutating their catalytic Ser to Ala—mimics antibiotic inhibition and activates the AmpR/AmpC pathway (27). Using a PBP4 S72A, PBP5 S64A double mutant (dacBC**), we tested whether impairing activation of AmpR/AmpC with TMP could potentiate the activity of cefotaxime (TAX), a drug that does not induce but is a substrate of AmpC. As expected, the dacBC** mutation conferred resistance, but the addition of TMP re-sensitized the mutant to TAX (Fig. 3D). Potentiation of TAX by TMP was lost in a dacBC** *ampG* triple mutant that lacks the inner membrane transporter required to import activating PG fragments, supporting our hypothesis that potentiation requires the canonical PG recycling pathway (Fig. 3D). A catalytically inactive AmpC S90A mutant also lacked potentiation (Fig. S3a), even though its expression was highly induced by FOX (Fig. S3b), confirming that AmpC activity is required for TMP synergy.

We reasoned that if TMP potentiated FOX activity by impairing *ampC* expression, then transcription from the *ampC* promoter should be reduced by TMP treatment. We created a luminescent *ampC* transcriptional reporter and saw strong induction of promoter activity following FOX treatment (Fig. 3E). Adding increasing amounts of TMP in addition to FOX led to a dose-dependent reduction in luminescence. The observed decrease in *ampC* promoter activity following TMP treatment implied that whole-cell AmpC activity would also be reduced. We grew cells with FOX (to induce AmpC) alone or in combination with increasing concentrations of TMP, then measured AmpC activity using nitrocefin, a chromogenic β-lactamase substrate (30). As predicted, we saw a dose-dependent decrease in nitrocefin hydrolysis in the presence of TMP vs the FOX-only control (Fig. 3F). To rule out the possibility that TMP was a direct inhibitor of AmpC activity, we performed the nitrocefin assay *in vitro* with purified AmpC and TMP and saw no inhibition (Fig. S4). These data support the hypothesis that anti-folate treatment blocks activation of the classic AmpR/AmpC response, increasing the susceptibility of the cells to β-lactams that are normally degraded by AmpC.

### Determinants of the TMP-FOS interaction

We next examined the mechanism of TMP-FOS potentiation. Unlike β-lactams, FOS inactivates a single cytoplasmic target (MurA), the first committed step of PG synthesis. The physiological consequences of inactivating a single enzyme can be modeled *in silico* using genome-scale metabolic reconstruction (GEMs) and predicted with constraint-based flux balance analysis (FBA) using COBRA (31). To determine whether TMP/SUL potentiation of FOS could be predicted from known *P. aeruginosa* physiology, we modeled possible impacts on metabolism using an established *P. aeruginosa* GEM and the COBRA FBA (32). The model could predict the well-established potentiation by the combination of TMP and SUL (Fig. S5a), but could not predict the TMP/SUL-FOS interaction (Fig. S5b and c). This failure suggests that the latter interaction results from an aspect of cell physiology—possibly PG recycling—that is not adequately modeled by FBA.

**Fig 4:**
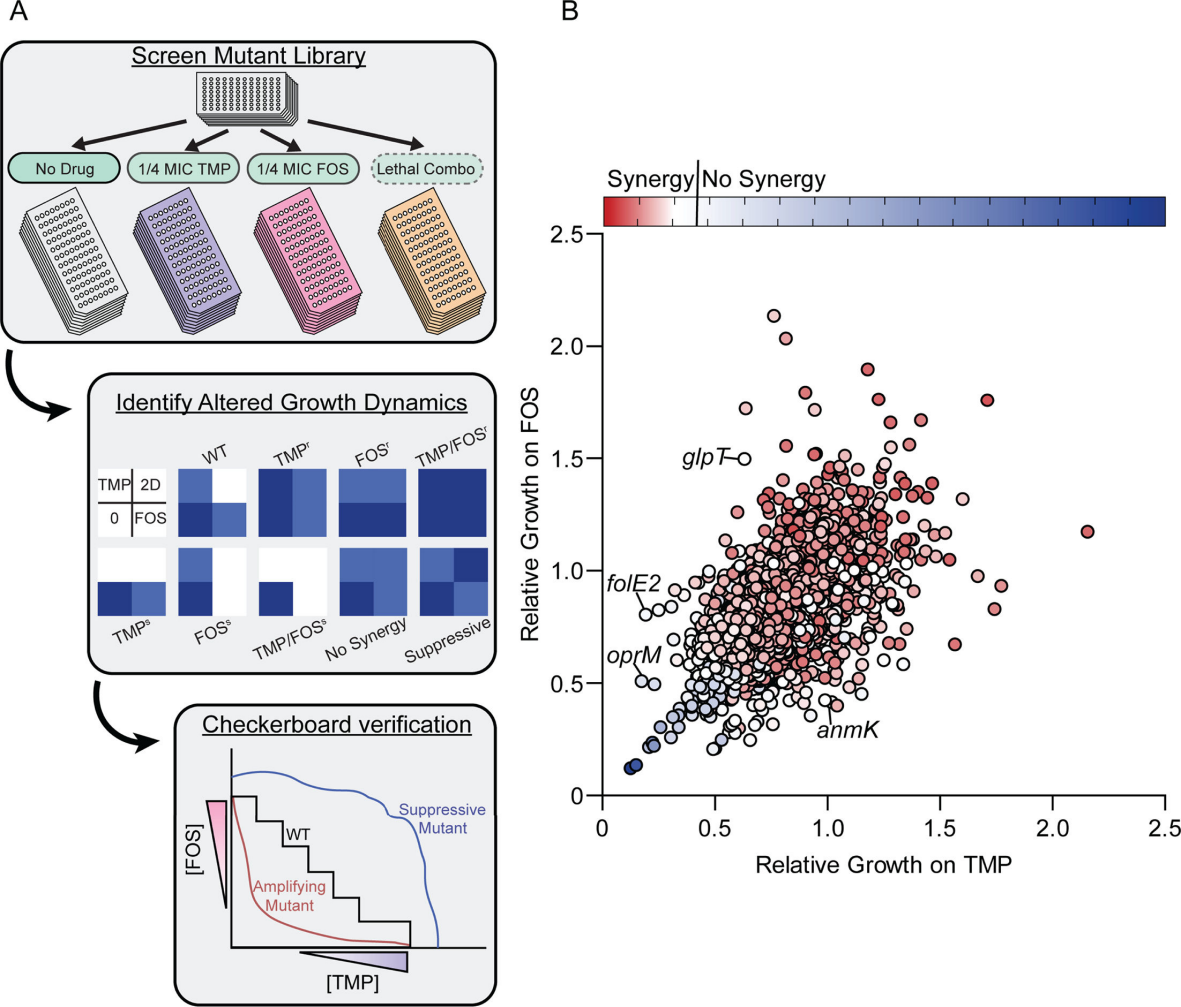
A genome-wide screen of TMP-FOS interaction determinants. (**A**) Schematic outlining the mutant library screen, analysis, and follow-up workflow. Top: four conditions on which the library was pinned. Middle: predicted outcomes for mutants in the screen. “2D” indicates the two-drug treated condition, while “s” and “r” indicate hypersensitive or resistant, respectively. Blue represents growth. Bottom: predicted outcomes for the wild type and mutants on a checkerboard assay. (**B**) A scatter plot showing the results of the PA14 library screen. Normalized growth relative to the untreated control for the TMP (*X*-axis)- or FOS (*Y*-axis)-only conditions are plotted on the axes. Red and blue indicate synergy or no synergy, respectively, while color intensity indicates the degree of synergy. Data points for select mutants that internally validated the screen are labeled.

FOS did not potentiate TMP or SUL against the hypersensitive *oprF* mutant, suggesting that loss of *oprF* and FOS treatment have overlapping effects on anti-folate susceptibility (Fig. S6). Because OprF has a number of predicted roles (33), its inability to modulate TMP-FOS potentiation was not particularly informative. Therefore, we sought to identify additional mutants with changes in TMP-FOS synergy. To do so, we screened an ordered PA14 transposon mutant library (34) at 1536-colony density in four conditions: no drug, sub-MIC TMP, sub-MIC FOS, or a synergistically lethal combination of the two drugs (Fig. 4 and Fig. S7a). A subset of mutants grew on the combination plate, suggesting a loss of synergy (Fig. S7b). A hit was defined as a mutant whose actual growth on the combination plate exceeded its expected growth (actual/expected > 1), as calculated from its growth on the individual antibiotic plates. Complete results for the full Tn library in the four screening conditions, with separate lists of all hits, plus those at 2 and 3 standard deviations from the mean, are provided in Table S2. We also identified mutants that were hypersusceptible to one or the other antibiotic (i.e., failing to grow on sub-MIC single compound plates in addition to the combination plate), leading to a loss of synergy. Providing internal validation of the screen, we identified mutants with known changes in susceptibility to TMP or FOS, including *anmK* (35), *oprM* (36), *folE2* (37), and *glpT* (26) (Fig. 4B), as well as *oprF*. Interestingly, several purine biosynthesis mutants, including *purN, purF, purL, purC,* and *purD,* showed increased sensitivity to the TMP/FOS combination. We selected eight purine biosynthesis mutants, including the five above, from the transposon library for follow-up assays. Notably, *purC* and *purM* mutants were ~8 and 4 times more sensitive to TMP than wild type (Fig. S8). *purD* and *purN* mutants were slightly more sensitive to the TMP/FOS combination, but the *purM* mutant was strikingly ~4 times more resistant to FOS. These data suggest that the purine biosynthesis pathway underpins the TMP-FOS interaction, but clarifying the nature of this connection will require additional studies.

### Disruption of PG recycling by TMP

We recognized that aberrant PG recycling caused by anti-folate treatment could be a shared determinant of sensitivity to both FOS and FOX in *P. aeruginosa* (38). We reasoned that treatment with TMP might change the abundance of specific soluble PG recycling fragments and used LC-MS to measure soluble PG species. TMP treatment significantly increased the abundance of GlcNAc-anhMurNAc and decreased the levels of anhMurNAc (Fig. S9), suggesting a possible block in PG recycling at the stage of cleaving the disaccharide moiety. Disaccharides lacking a stem peptide are the products of amidase activity and act as substrates for the NagZ β-N-acetylglucosaminidase. AmpD amidase activity is negatively correlated with AmpC induction (28), while NagZ activity is required for FOS resistance (39), so the accumulation of the disaccharide is consistent with factors reported to increase FOX and FOS sensitivity.

### A novel dual inhibitor overcomes meropenem resistance by targeting FolP and NDM-1

**Fig 5:**
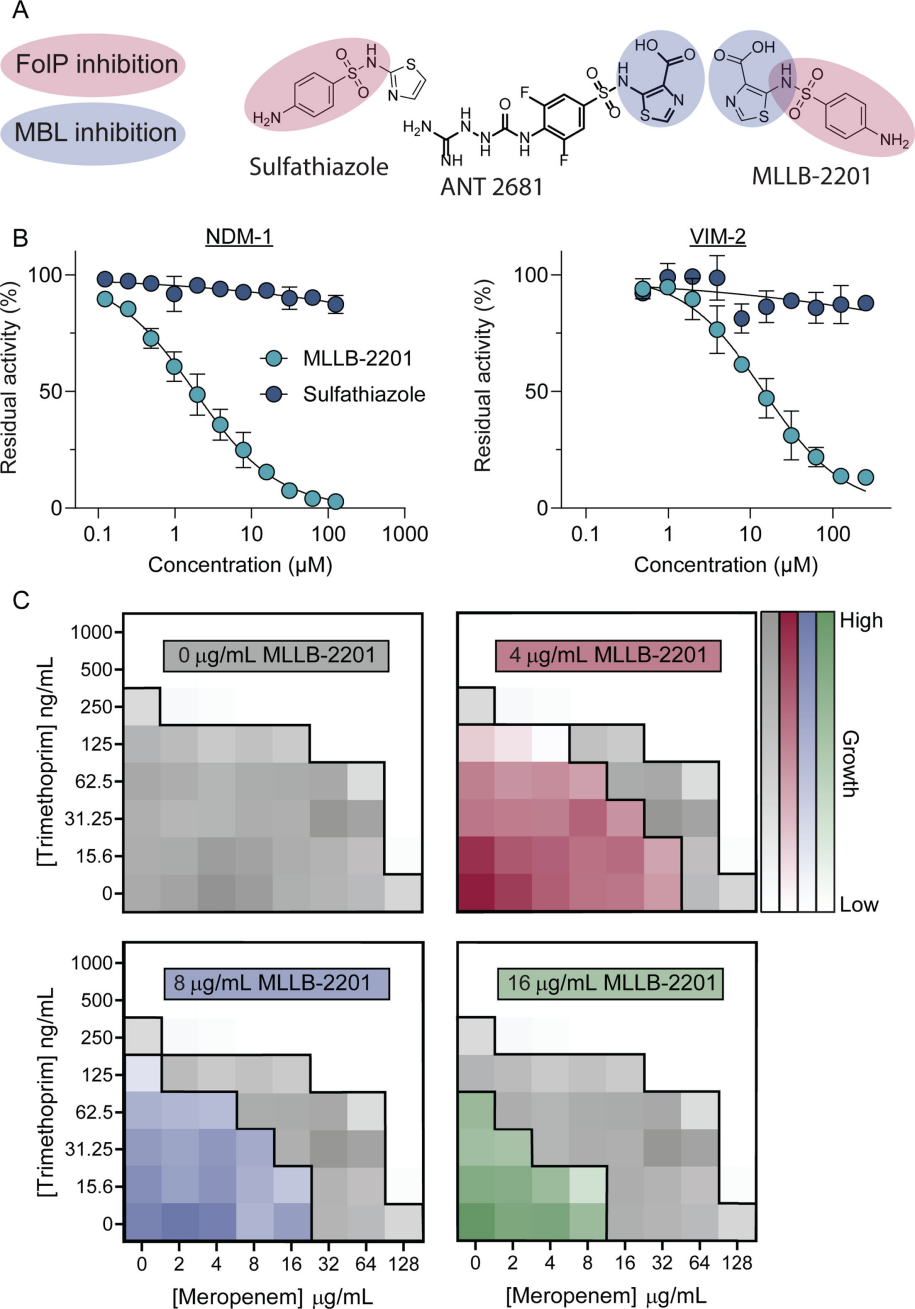
A dual-active inhibitor of metallo-β-lactamases overcomes resistance to meropenem via triple synergy. (**A**) Chemical warheads responsible for FolP and metallo-β-lactamase inhibition are highlighted in red and blue, respectively. (**B**) Effects of MLLB-2201 (light blue circles) and sulfathiazole (dark blue circles) on NDM-1 (left) and VIM-2 (right) hydrolysis of meropenem. Increasing concentrations of each inhibitor are plotted on the *X*-axis, while the residual enzyme activity is plotted on the *Y*-axis as a percent of the uninhibited enzyme activity. Two biological replicates were performed, each with two technical replicates. A representative biological replicate is shown, with the mean of the technical replicates plotted as a colored circle and the standard error of the mean shown. To calculate IC_50_ values, curves were fitted to each data series. (**C**) Three-way checkerboards showing the effects of trimethoprim, meropenem, and MLLB-2201 on *E. coli* expressing NDM-1. The concentrations of trimethoprim and meropenem are shown on the left and bottom axes, respectively. The concentration of the 3rd compound, MLLB-2201, is indicated within each checkerboard. The growth values for the three triple-combination checkerboards are overlayed in colored squares on top of the trimethoprim and meropenem-only checkerboard data to illustrate the effects of the MLLB-2201 addition. Checkerboard assays were repeated in biological duplicates and data from a representative replicate are shown.

While considering how to exploit the connections between folate and PG metabolism in the design of new antibiotics, we noticed the remarkable structural resemblance of ANT-2681—a recently developed inhibitor of metallo-β-lactamases (MBL) that degrade β-lactams—to the anti-folate drug, sulfathiazole (40) (Fig. 5A). ANT-2681 contains the core 2-sulfanilamidothiazole of sulfathiazole plus additional substituents, including a carboxylate moiety that coordinates active-site zinc ions to inhibit MBL activity (PDB 6ZGM) (40). The key structural feature of sulfathiazole is the sulfanilamide moiety that competes with PABA for the FolP-binding pocket. Available structures of FolP bound to sulfathiazole (PDB 3TYE) showed that the variable ring of sulfonamides sits outside the binding pocket and thus might tolerate substitution on the thiazole moiety (41).

Leveraging this information, we designed and synthesized MLLB-2201, a carboxylate-containing sulfonamide with potential dual activity against FolP and MBLs (Fig. 5A). We hypothesized that by inhibiting both FolP and MBLs, MLLB-2201 should synergize with β-lactams and TMP. As predicted based on retention of the ANT-2681 core, MLLB-2201 was a low micromolar inhibitor of the MBLs NDM-1 and VIM-2, with IC50 values of 1.8 and 14.5 µM, respectively (Fig. 5B). Despite having a higher IC_50_ than ANT-2681 (40), MLLB-2201 restored meropenem activity against an *E. coli* strain expressing NDM-1, whereas sulfathiazole could not (Fig. S10a). When we tested MLLB-2201’s ability to inhibit folate synthesis using a model Gram-positive (to avoid permeability issues common with Gram negatives), we saw minimal growth inhibition of methicillin-resistant *Staphylococcus aureus,* although it synergized with TMP (Fig. S10b), suggesting that it has some FolP inhibitory activity. Finally, when MLLB-2201 was tested in combination with TMP and meropenem against *E. coli* expressing NDM-1, we observed three-way synergy (Fig. 5C). MLLB-2201 (16 µg/mL) in combination with TMP (31.25 ng/mL) and meropenem (4 µg/mL) inhibited growth of this strain, representing 16-fold and 64-fold reductions in the single-antibiotic MICs.

## DISCUSSION

Tetrahydrofolate plays a central role in one-carbon metabolism and the synthesis of critical metabolites, including thymidylate, purines, methionine, and glycine/serine. Therefore, disrupting folate biosynthesis is an effective way to kill bacteria. Because folate inhibition has multiple consequences for metabolism, its specific impacts on other pathways can be challenging to study. Using a combination of bioinformatics, microscopy, and chemical genetics, we characterized the relationship between folate and PG in *P. aeruginosa*. Our data point to a requirement for folate metabolism in the maintenance of normal cell wall turnover and induction of *ampC* expression through the AmpR pathway.

Our data suggest that anti-folates impair PG recycling, explaining why TMP uniquely potentiated FOX and FOS in our initial PG-inhibitor interaction screen. Using inactive point mutants of PBP4 and PBP5 that accumulate the sentinel anhMurNAc-pentapeptide recycling product and activate AmpR/AmpC, we showed that TMP suppresses AmpC expression to potentiate the activity of non-inducing β-lactams. This potentiation required AmpG, the muropeptide permease, and the regulator AmpR, suggesting that the sentinel recycling product must enter the cytoplasm and activate AmpR by the canonical mechanism. Disrupting PG recycling is an established strategy to potentiate FOS in *P. aeruginosa*, as its unusual ability to salvage MurNAc can bypass the inhibition of MurA and *de novo* MurNAc synthesis (39, 42). In the salvage pathway, AnhMurNAc released from recycled PG fragments by NagZ is converted to MurNAc-6-P by AnmK. After dephosphorylation at the 6 position by MupP, sugar kinase AmgK adds a new phosphate at the 1 position, and uridylyltransferase MurU generates UDP-MurNAc. Our LC-MS analysis showed that TMP significantly increased the abundance of GlcNAc-anhMurNAc and decreased the abundance of free anhMurNAc, suggesting a possible bottleneck at the NagZ step. Alternatively, an increase in RlpA activity—a lytic transglycosylase that specifically cleaves stemless PG and liberates it for recycling (43)—could generate excess GlcNAc-anhMurNAc. Notably, disruption of PG recycling compromises the fitness of *P. aeruginosa in vivo*, suggesting treatment with anti-folates could impact virulence (44). Therefore, the overall impact of anti-folate treatment could be multifactoral, both inhibiting the synthesis of key biosynthetic precursors and limiting access to recycled PG metabolites that could otherwise help bacteria to survive via metabolic detours.

Despite defining the interaction of anti-folates with specific PG inhibitors, we have yet to precisely identify which downstream effects of folate inhibition compromise PG metabolism. Our PA14 transposon library screen pointed to impacts on purine biosynthesis. Previous work in *B. subtilis* showed that depleting purines but not thymidylate (both folate-dependent metabolites) caused a large decrease in PG turnover (45, 46). How the cells might sense purine depletion and concomitantly decrease the rate of PG turnover remains unclear. To uncover more factors involved in regulating PG turnover, the dacBC** mutant with constitutively elevated *ampC* expression could be used. Mutations or small molecules that decrease *ampC* promoter activity in that background are likely to be compromised in PG recycling. Such a screen might uncover new factors affecting PG turnover and reveal novel ways to increase the sensitivity of *P. aeruginosa* to AmpC-inducing β-lactams. Here we identified TMP as one such inhibitor that reduces degradation of β-lactams by AmpC, opening a new avenue for possible β-lactam adjuvant development.

Building on the finding that folate inhibitors can potentiate β-lactams in *P. aeruginosa*, we designed MLLB-2201 as a dual inhibitor of metallo-β-lactamases and FolP, the target of sulfonamides. In theory, this compound could achieve three-way potentiation with a β-lactam and TMP against *P. aeruginosa*. (i) TMP and MLLB-2201 could synergize by inhibiting different steps in folate metabolism. (ii) MLLB-2201 could block β-lactam degradation by metallo-β-lactamases using its MLB inhibitor scaffold. (iii) TMP and MLLB-2201 could decrease *ampC* induction, thus reducing β-lactam hydrolysis by AmpC. While we achieved some of these effects, *P. aeruginosa* remained largely unaffected by MLLB-2201 alone, potentially due to its notorious impermeability (47). A medicinal chemistry effort is currently underway to increase the potency of MLLB-2201, with a focus on improving FolP inhibition and anti-*Pseudomonas* activity.

Folate and PG metabolism must be carefully coordinated with other cellular processes for balanced growth. For example, the tailoring of PG peptide stems by D,D-carboxypeptidases, the targets of FOX, limits PG crosslinking and regulates the activity of the Bam β-barrel assembly complex (48). MurA and LpxC catalyze the first committed steps of PG and lipopolysaccharide (LPS) biosynthesis, respectively, and in *P. aeruginosa*, MurA activates LpxC through a physical interaction that ensures the balanced consumption of UDP-GlcNAc by the PG and LPS biosynthetic pathways (49). These and other intersections between key pathways show that disruption of one can create potential vulnerabilities in others. Our data suggest that the folate and PG pathways are more intimately connected than previously appreciated and that targeting their nexus could open new avenues for drug development.

## MATERIALS AND METHODS

### Bacterial strains and growth conditions

All bacterial strains used in this study are listed in Table S1. Strains were stored at −80°C in 15% glycerol stocks. For experiments, overnight cultures were grown in lysogeny broth (LB) at 37°C with shaking at 200 rpm. For plasmid maintenance, antibiotics were added at the following concentrations: gentamicin at 15 µg/mL for *Escherichia coli* or 30 µg/mL for *Pseudomonas aeruginosa*, ampicillin at 100 µg/mL for *E. coli*, and carbenicillin at 200 µg/mL for *P. aeruginosa*.

### Plasmid and strain construction

Plasmids were constructed using standard molecular biology techniques and verified by DNA sequencing. Primers and plasmids used in this study are listed in Table S1. pMS403 was derived from pMS402Gm by digesting pMS402 and pPS856 with PstI, then isolating the gentamicin resistance cassette from the pPS856 digest reaction and using T4 ligase to insert the gentamicin cassette into pMS402. Next, pMS402Gm was digested at a BsiWI site within the *DHFRII* gene, the overhangs were filled in using Klenow, and the resulting blunt ends were rejoined by ligation with T4 ligase and transformed into *E. coli* DH5α. Transformants were selected on Gent15 and tested for trimethoprim sensitivity to confirm *DHFRII* inactivation. pMS403 (P*ampC*) was created by PCR amplifying the *ampC* promoter with P*ampC* Fwd and P*ampC* Rvs (Table S1), then digesting both pMS403 and the purified PCR product with BamHI and XbaI, and ligating the two purified digest products with T4 ligase. The ligation product was transformed into *E. coli* DH5α.

pUCP20 (*DHFRII*) was created by PCR amplifying the *DHFRII* gene from the pMS402 backbone with *DHFRII* Fwd and *DHFRII* Rvs primers (Table S1). Next, pUCP20 and the purified PCR product were digested with EcoRI and HindIII, the products were ligated with T4 ligase, and then transformed into *E. coli* DH5α.

pEX18Gm (*ampC*), pEX18Gm (*ampC* S90A), and pEX18Gm (*ampG*) were constructed by digesting gBlock inserts in pUC57 (Genscript) with EcoRI and HindIII (or SacI for *ampG*). The inserts were gel purified and ligated using T4 ligase into pEX18Gm digested with EcoRI and HindIII/SacI. The ligations were transformed into *E. coli* DH5α. pEX18Gm (*ampR*) was constructed by amplifying 500 bp regions of chromosomal DNA from PAO1 that flank the 5′ and 3′ ends of *ampR* using two PCRs containing the d*ampR* Up Fwd and d*ampR* Up Rvs primers, or the d*ampR* Dwn Fwd and d*ampR* Dwn Rvs primers (Table S1). The purified PCR products from these reactions were combined and used as templates for overlap extension PCR with the d*ampR* Up Fwd and d*ampR* Dwn Rvs primers, and the purified PCR product was digested with BamHI and HindIII. The purified digest product was ligated into linearized pEX18Gm (digested with BamHI and HindIII) using T4 ligase. The ligations were transformed into *E. coli* DH5α.

pMS403- and pUCP20-based plasmids were introduced to *P. aeruginosa* by electroporation. pEX18Gm-based plasmids were first introduced into *E. coli* SM10 by electroporation, then *E. coli* SM10 was used to transfer the plasmid into *P. aeruginosa* by conjugation. *P. aeruginosa* cells containing the plasmid were selected for on *Pseudomonas* isolation agar (PIA) containing 100 µg/mL of gentamicin to select for the first recombination event. Colonies from the PIA Gent100 plates were streaked onto LB 5% sucrose no NaCl agar plates and incubated at 30°C to select for a second recombination event. Colonies from the LB sucrose plates were patched onto LB and LB Gent30 plates. Patches that grew on LB but not LB Gent30 plates were tested for loss of intrinsic ampicillin resistance, which indicates loss of AmpC activity. Mutants were confirmed with PCR.

Strains containing the dacB*/dacC* catalytically inactive point mutations were constructed by site-directed mutagenesis of PAO1 wild-type alleles. The dacB* primer included a TCG→GCG mutation that converted the serine 72 codon to an alanine, while the dacC* primer included an AGC→GCG mutation that converted the serine 64 codon to an alanine. These mutant alleles were crossed into the chromosome of PAO1 using allelic exchange with the pEX18Gm plasmid. Mutants were confirmed by sequencing mutant alleles amplified by PCR.

### MIC assays

MIC assays were performed in 96-well plates. Overnight cultures were diluted 1:100 into fresh LB and grown to OD_600_ of ~0.1–0.3. Cultures were normalized to OD_600_ of 0.1, diluted 1:500 into fresh LB, and 150 µL of diluted cultures were added to wells. Antibiotics were added in serial twofold dilutions. Plates were incubated at 37°C with shaking, and growth (OD_600_) was measured after 18 h using a plate reader (Multiskan Go, Thermo Fisher Scientific).

### Microscopy

Overnight cultures were diluted 1:100 in LB with or without TMP at the indicated concentrations and incubated until an OD_600_ of ~0.4. All cultures were normalized to an OD_600_ of 0.3, then 1 mL of each culture was pelleted, washed with 1 mL of sterile PBS, and then resuspended in 100 µL of sterile PBS. Four microliters of the resuspended cells was spotted on 1.5% M9 + glucose agarose pads on glass slides and covered with coverslips. Cells were imaged with a Nikon Ti-2 Eclipse inverted confocal microscope using a 60× oil immersion objective lens. Identical settings were used for all micrographs captured.

### Checkerboard (synergy) assays

Checkerboard assays were performed by adding serial dilutions of two antibiotics in 96-well plates as described previously (50). Overnight cultures were normalized to OD_600_ of 0.1, diluted 1:500, and 150 µL of the diluted cultures were added to wells. Antibiotic combinations were tested for synergy, and growth (OD_600_) was measured after 18 hours at 37°C using a plate reader. Fractional inhibitory concentration index (FICI) values were calculated to determine drug synergy. FICI is calculated with the equation below:


$$\frac{{\left[A\right]}_{AB}}{{\left[A\right]}_{A}}+\frac{{\left[B\right]}_{AB}}{{\left[B\right]}_{B}}=FICI$$

where [A] and [B] are the concentrations of compound A or compound B at a given well where no growth is observed, and the subscript (A, B, or AB) indicates whether A or B was added alone or in combination. An upper limit of 0.5 is used to determine synergy.

### Bioinformatic analyses

To investigate synteny between folate and peptidoglycan biosynthesis genes, we performed tblastn searches against a custom database of bacterial genomes obtained from the Genome Taxonomy Database (GTDB) (51). Protein sequences for FolP and GlmM from *Pseudomonas aeruginosa* PAO1 were used as query sequences. Genomic distances between *folP* and *glmM* were calculated for all genomes with hits for both genes. We targeted proteobacterial families with 100 or more genomes labeled as complete in the RefSeq database. This subset, encompassing a total of 939 genomes, was used to construct a phylogenetic tree. Phylogenetic trees of representative genomes were constructed using FastTree2 (52), and visualized using the Interactive Tree of Life (iTOL) (53).

### Luminescent promoter-reporter assays

Overnight cultures of PAO1 containing pMS402(empty) or pMS402(P*ampC*) were made by inoculating 3 mL of LB Gent30 from frozen stocks and were incubated at 37°C with shaking. Subcultures were made by transferring 120 µL of the overnights into 3 mL of LB Gent30 and incubated at 37°C with shaking for ~2 hours until an OD_600_ of ~0.1–0.3 was reached. Cultures were normalized to an OD_600_ of 0.1, then diluted 1:500 in fresh LB Gent30. Assays were prepared in white-walled 96-well plates with clear bottoms (Corning). Two microliters of dilutions of trimethoprim was added across rows A–E at the indicated concentrations. Cefoxitin was added to the wells in rows A–F at a final concentration of 50 µg/mL. A DMSO vehicle control was added to wells in rows G and H. Afterward, 148 µL of the diluted culture was added to each well of the plate, except row H, which served as a sterility control and received 148 µL of LB Gent 30. The plate was incubated at 37°C with continuous double orbital shaking for 16 h in a Synergy Neo (Biotek) plate reader. Growth (OD_600_) and luminescence (luminescence fiber) measurements were taken every 15 min and promoter activity at 8 h was graphed. Relative luminescence units (RLU) were calculated by dividing each well’s luminescence value by its growth at the corresponding time point.

### Determination of β-lactamase activity

Whole-cell AmpC β-lactamase activity was determined using the nitrocefin hydrolysis assay (54). Overnight cultures were diluted 1:100 into fresh LB and grown to mid-log phase. Cells were pelleted by centrifugation and washed with 50 mM sodium phosphate buffer (pH 7.4). The washed cells were then resuspended in sodium phosphate buffer and lysed by sonication. Cell debris was removed by centrifugation, and the supernatant was used for the nitrocefin hydrolysis assay. Nitrocefin was added at a final concentration of 50 µM, and absorbance at 490 nm was measured every 15 s over 10 min using a spectrophotometer (Multiskan Go, Thermo Fisher). Data were analyzed by comparing the rate of nitrocefin hydrolysis between control and antibiotic-treated samples. *In vitro* AmpC activity assays were performed using purified AmpC from *Pseudomonas aeruginosa* (Sigma-Aldrich). The purified enzyme was incubated with nitrocefin in the presence or absence of TMP, and the rate of hydrolysis was measured at 490 nm. IC50 values for TMP were determined by fitting curves to the data.

*In vitro* metallo β-lactamase activity was measured using NDM-1 (5 nM) or VIM-2 (50 nM) incubated in reaction buffer (25 mM HEPES-NaOH, 10 µM ZnSO_4_, pH 7.5) containing varying amounts of inhibitor (1–500 μM) and incubated for 5 min at room temperature. Residual enzyme activity was determined by measuring β-lactam hydrolysis spectrophotometrically at 300 nm by adding a saturating amount of meropenem (500 µM) to the reaction mixtures containing enzyme and inhibitor. β-Lactamase assays were performed in a clear flat-bottom 96-well plate at 25°C with a final assay volume of 200 µL and monitored with a BioTek Synergy H1 microplate reader over 10 min. All reactions were performed in duplicate unless otherwise stated.

### Flux balance analysis

The iPAO1 genome-scale metabolic model developed by Zhu et al. (32) was imported into Matlab R2020a (MathWorks) and the Cobra toolbox (Version 3.0) (31) was used to perform FBA with a Gurobi mathematical solver (Version 9.0.2). FBA-Div was used to simulate antibiotic treatment (55), where the substrates of inhibited reactions are diverted to a waste reaction. The iPAO1 model contains an irreversible and reversible reaction for FolP; therefore, to inhibit both reactions, the irreversible reaction (rxn02201) was removed from the model, and flux through the reversible reaction (rxn02200) was reduced, and the substrate (dihydropteroate) was diverted to a waste reaction. To simulate a dilution range of antibiotic treatment, the optimal flux for each reaction was determined under no reaction inhibition, then the flux rate was simulated at 20% intervals from 0%–100% of the optimal flux rate. The calculated biomass production rate at the steady state for each interval was determined in single and double reaction inhibitions to generate growth values that were used to create a checkerboard. Rich media growth conditions were assumed for the metabolic modeling.

### Chemical-genetic screening

Rectangular plates with 25 mL of LB 1.5% agar containing no antibiotic, 32 µg/mL fosfomycin, 32 µg/mL trimethoprim, or both antibiotics at 32 µg/mL, were poured and allowed to dry overnight. An ordered PA14 transposon mutant library (34) was pinned from source plates in 1536 colony density onto the rectangular agar plates using a ROTOR HDA robotic colony replicator (Singer Instruments). The screen was performed in duplicates using different source plates. Plates were incubated for 18 h at 37^o^C, then imaged using a Phenobooth imaging system (Singer Instruments) using the transmissive light mode. Images were further processed in FIJI (Image J) as described previously (56). Briefly, the light absorbed by each colony was converted into an integrated density value. Integrated densities were then normalized for plate position effects and the relative growth was determined by comparing to the untreated control. The relative synergy score was calculated by multiplying the normalized growth scores for the single-drug TMP and FOS treatment conditions to obtain the theoretically expected growth in the two-drug treatment condition. The observed two-drug treatment growth was divided by the expected two-drug treatment growth to determine the relative synergy score. The raw data for this screen are provided as an Excel file, Table S2. Mutants with synergy scores above 1 are provided as a hit list in a separate tab, with additional tabs containing lists of those mutants at 2 and 3 standard deviations above the mean relative synergy score.

### LC-MS analysis of soluble peptidoglycan fragments

Soluble muropeptides were prepared according to Weaver et al. (57) with minor modifications. Briefly, 50 mL flasks of LB were inoculated with 0.5 mL of an overnight culture. Flasks were prepared in technical duplicate with antibiotics added at the indicated concentrations. Cultures were incubated at 37°C while shaking until an OD_600_ of ~0.4 was reached. Then, flasks were immediately placed on ice, cultures were normalized to an OD_600_ of 0.3, and 40 mL were transferred to pre-chilled 50 mL Falcon tubes. The cultures were centrifuged at 5,030 × *g* and 4°C for 20 min on an Avanti J-26 XPI (Beckman-Coulter) centrifuge (JS 5.3 rotor). The supernatant was decanted, and cell pellets were resuspended in 1 mL ice-cold 0.9% NaCl, washed twice with 1 mL 0.9% NaCl, and resuspended in 1 mL of sterile nuclease-free water. The resuspended cells were boiled for 30 min to lyse them, and centrifuged for 15 min at 21 000 × *g* in a benchtop centrifuge to pellet the cell debris. The supernatant was passed through a 0.2 µm filter and frozen at −80°C. Samples were concentrated as needed under vacuum using a lyophilizer (Virtis), dissolved in sterile nuclease-free water, and pH adjusted to ~3 using formic acid. After adjusting the pH, samples were centrifuged for 10 min at 21,000 × *g* in a benchtop centrifuge to pellet any precipitate.

Ten microliters of each sample was injected into an LC/Q-TOF (Agilent 6546) and separated using an Eclipse Plus C18 column (Agilent, 95 Å pore size, 2.1 × 100 mm, 1.8 µm) at 50°C. Separation of PG species was achieved using a linear gradient of buffer A (water + 0.1% formic acid) to buffer B (acetonitrile + 0.1% formic acid) over a 56 minute run time with a 0.4 mL/minute flow rate. The Q-TOF was run in negative ionization mode at 4,000 V capillary voltage, 300°C source temperature, 300°C sheath gas temperature, and a scan range of 100–1,700 *m/z*. Data acquisition and analysis were performed using the Agilent MassHunter qualitative analysis (v10.0) software. Extracted ion chromatograms were manually curated using theoretical muropeptide masses. The selected muropeptides were based on *m/z* values of 595.6639 for UDP-MurNAc-AEmAA (doubly charged species), 524.6263 for UDP-MurNAc-AEm (doubly charged species), 477.1726 for GlcNAc-anhMurNAc (singly charged species), and 274.0932 for anhMurNAc (singly charged species).

### Synthesis of MLLB-2201

See supplemental information for synthesis details and NMR validation data for MLLB-2201.

### Construction of graphs, structures, and statistical analysis

All graphs and checkerboards were created using GraphPad (Prism, Version 10), and statistical analyses were also performed in GraphPad. The graph in Fig. 1C was created using R and the tree in Fig. 1D was created using iTOL. The structures shown in Fig. 1A were downloaded from the Protein Data Bank and modeled in ChimeraX (Version 1.4) (58).

## SUPPLEMENTAL MATERIAL

Supplemental figures mbio.02984-24-s0001.pdf Figures S1-S10 and legend to Video S1.

Supplemental Information mbio.02984-24-s0002.pdf Captions to Tables S1 and S2, plus the methods and references for synthesis of MLLB-2201 and validation of the molecule's structure by NMR.

Table S1 mbio.02984-24-s0003.docx Strains and primers used for this study.

Table S2 mbio.02984-24-s0004.xlsx TMP-FOS synergy screen of the PA14 transposon library, including all raw data plus hit lists.

Video S1 mbio.02984-24-s0005.avi TMP treatment causes explosive cell lysis.

ASM does not own the copyrights to Supplemental Material that may be linked to, or accessed through, an article. The authors have granted ASM a non-exclusive, world-wide license to publish the Supplemental Material files. Please contact the corresponding author directly for reuse.
