# Supplementary material for: Perturbation of Pseudomonas aeruginosa peptidoglycan recycling by anti-folates and design of a dual-action inhibitor
Source: mBio. 2025 Jan 29;16(3):e02984-24. doi: 10.1128/mbio.02984-24 (PMC11898565; doi:10.1128/mbio.02984-24)
Supplement: Supplemental figures — Figures S1-S10 and legend to Video S1. [file mbio.02984-24-s0001.pdf]

Extended data for:

Perturbation of *Pseudomonas aeruginosa* peptidoglycan recycling by anti-folates and design of a dual-action inhibitor

Luke N. Yaeger[1], David Sychantha[1], Princeton Luong[1], Shahrokh Shekarriz[1], Océane Goncalves[2], Annamaria Dobrin[1], Michael R. Ranieri[1], Ryan P. Lamers[1], Hanjeong Harvey[1], George C. diCenzo[3], Michael Surette[1], Jean-Philippe Côté[2], Jakob Magolan[1], and Lori L. Burrows[1*]

[1]Department of Biochemistry and Biomedical Sciences, and the Michael G. DeGroote Institute for Infectious Disease Research, McMaster University, Hamilton, Ontario, Canada
[2]Département de Biologie, Université de Sherbrooke, Sherbrooke, Québec, Canada
[3]Department of Biology, Queen's University, Kingston, Ontario, Canada

***For correspondence:**
Dr. Lori Burrows, PhD FAAM FCAHS
lori.burrows@mcmaster.ca

TMP Treated Time Course.avi

Extended Data Movie 1 (supplied separately as .avi file). TMP treatment causes explosive cell lysis. A time lapse of TMP treated cells was created by capturing images every 15 seconds, compiling all images into an image stack, and exporting the stack as an .avi file at 5 frames per second. The round cell undergoes explosive cell lysis.

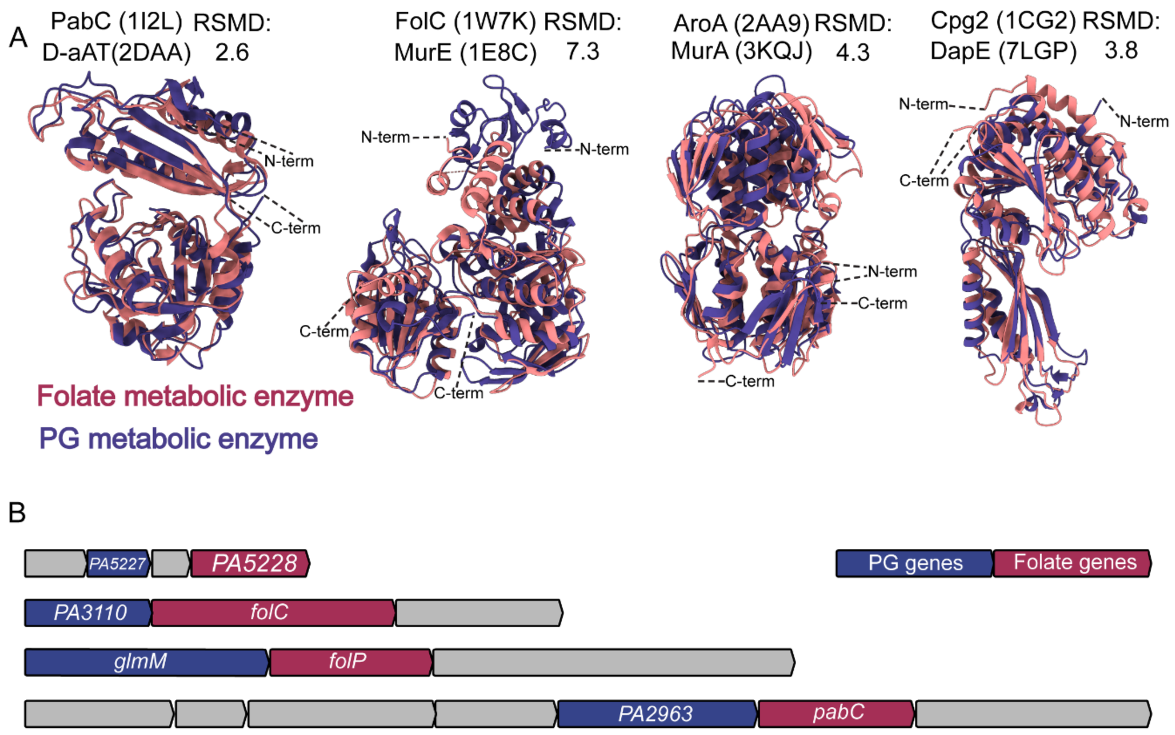

Extended Data Figure 1. Folate-PG structural and synteny relationships. A) A comparison of structural similarity between folate (orange) and PG (blue) metabolic enzymes. The structures were overlayed using the Matchmaker function in ChimeraX. Above each pair of structures are the protein names and PDB codes, where the top name and code corresponds to the folate protein, while the bottom name and code correspond to the PG protein. **B)** An illustration of the *P. aeruginosa* PAO1 operons containing folate and PG-related genes (shown in red and blue, respectively). Other genes within the predicted operons are shown in gray. The unnamed genes are labelled with their *P. aeruginosa* PAO1 locus tag. PA5227 encodes a ZapA homologue, PA5228 encodes a Fau homologue, PA3110 encodes a DedD homologue, and PA2963 encodes an MltG homologue.

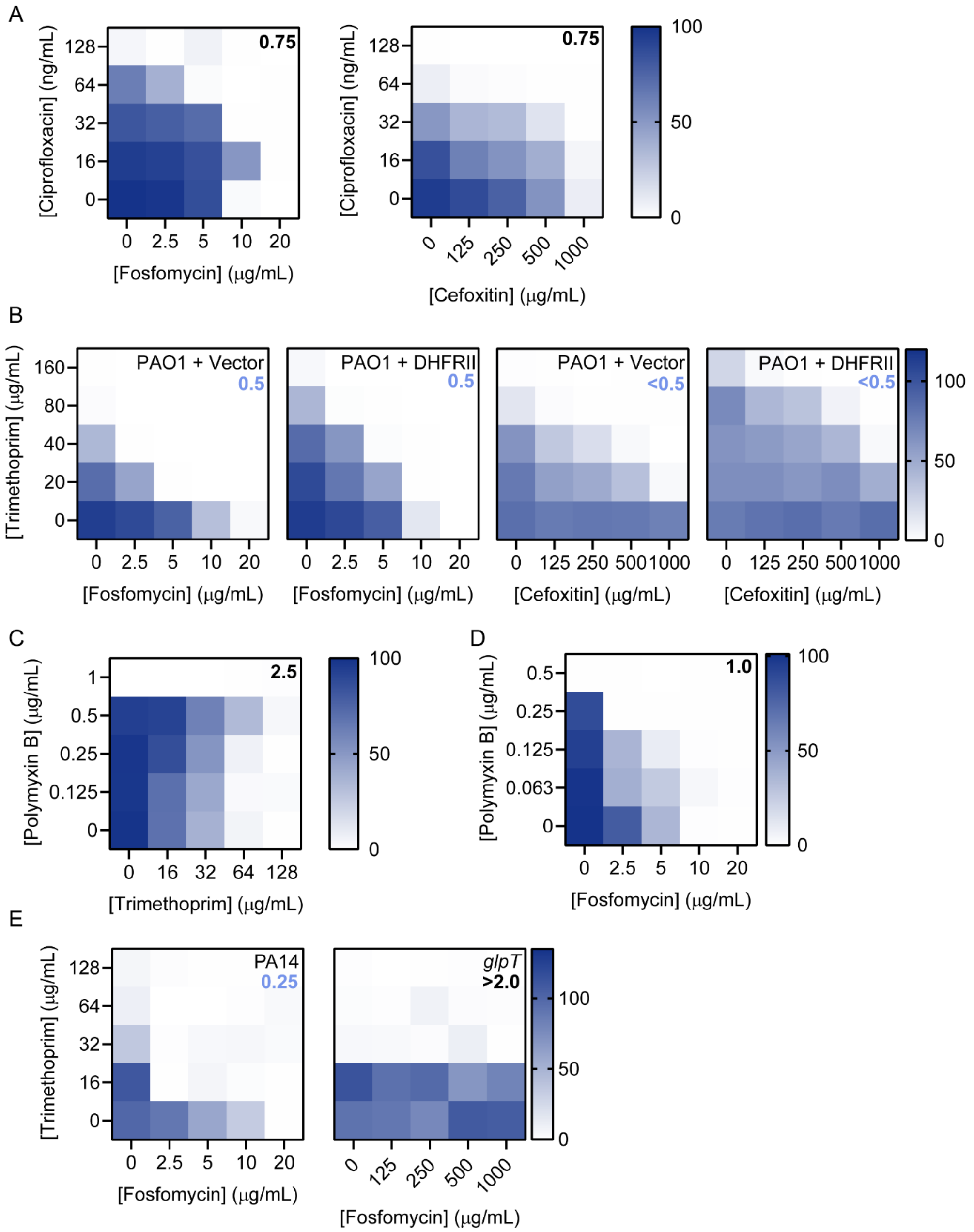

Extended Data Figure 2. TMP potentiates FOS and FOX through its primary MOA.
A-E) Checkerboard assays between two antibiotics (labelled on X- and Y-axes). Checkerboards were performed using an 8x8 concentration range and condensed into a 5x5 concentration range (labelled on axes) for each figure. The scale bar on the right indicates the amount of growth where white shows no growth and dark blue shows the highest growth. Growth is represented as a percent of the untreated control growth for each matched assay. Each assay was performed in biological duplicate and a representative replicate is shown. For B and E), the strains used for each assay are labelled in the top right corner. PAO1 was used for A, C, and D. FICIs are shown in the top right corner.

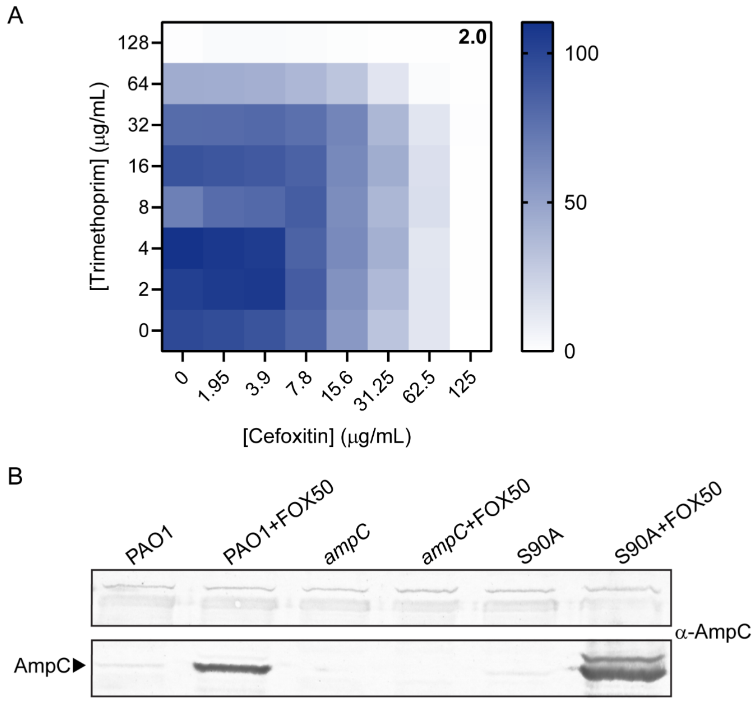

Extended Data Figure 3. AmpC activity is required for TMP potentiation of FOX. A) An 8x8 checkerboard assay measuring interactions between trimethoprim and cefoxitin using the *ampC* S90A strain. The colour gradient from white to blue indicates the growth as a percent of the untreated control and the legend is shown on the right. Assays were performed in biological duplicate and a representative replicate is shown. FICI is shown in the top right corner. **B)** Western blot for AmpC using whole cell lysates. FOX50 = 50 µg/mL of cefoxitin. The bottom panel shows bands corresponding to the molecular mass of AmpC (indicated with the arrowhead on the left). Note that the higher mass band in the S90A+FOX50 lane is likely unprocessed AmpC with the N-terminal signal peptide which accumulates due to high levels of induction. The top panel shows non-specific bands used as a loading control. A representative blot for two independently prepared biological replicates is shown.

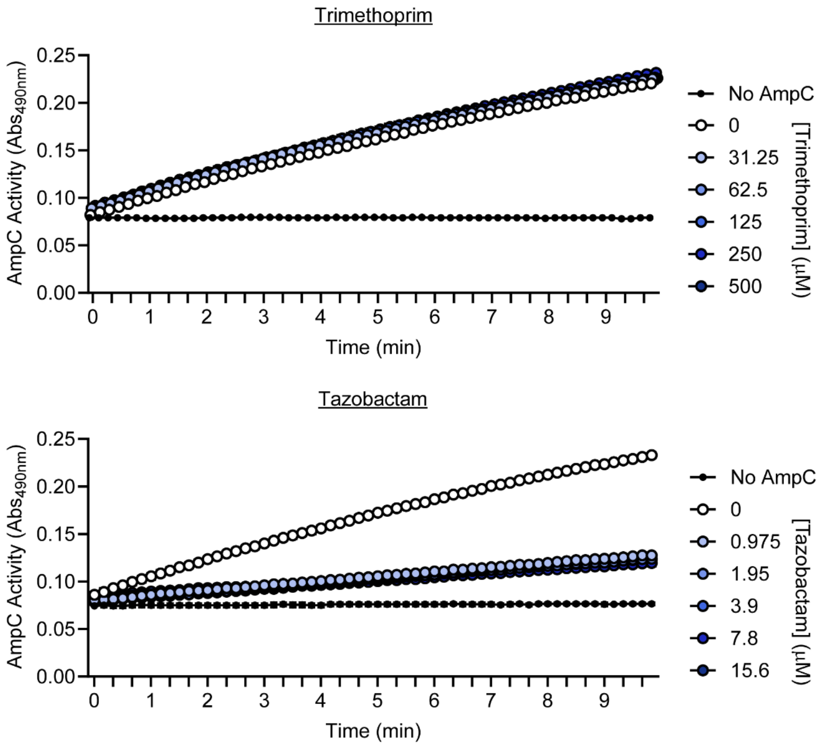

Extended Data Figure 4. Trimethoprim does not directly inhibit AmpC activity. The effect of trimethoprim (top) and the β-lactamase inhibitor Tazobactam (bottom) on activity of purified AmpC was measured via nitrocefin hydrolysis over time (X-axis). Hydrolyzed nitrocefin absorbs light at 490 nm and absorbance values are plotted along the Y-axis. Concentrations of each compound are shown on the right. Circles are the mean of two technical replicates, and the error bars indicate the standard error of the mean. Experiments were repeated in biological duplicate and a representative replicate is shown.

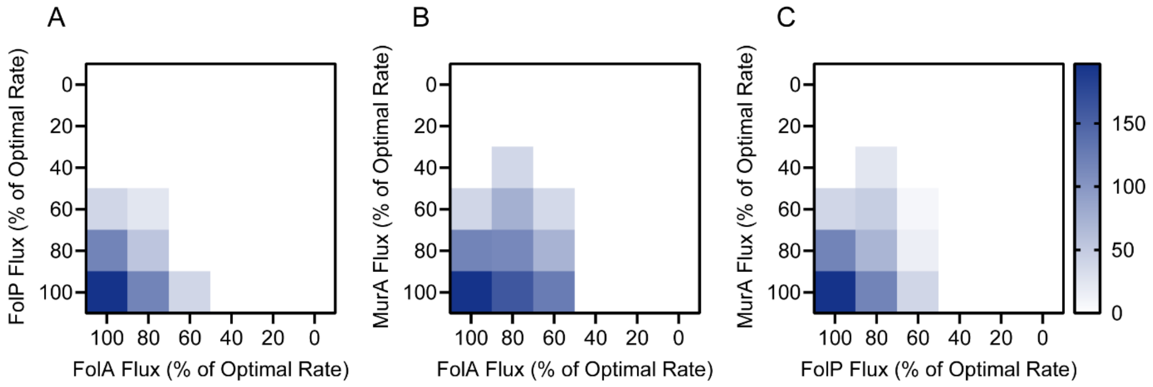

Extended Data Figure 5. Antifolate-fosfomycin interactions are not predicted by *in silico* modelling. A-C) Heatmaps showing the combinatorial effects of reducing flux through FolP, FolA, and MurA in an *in silico* genome scale metabolic model. The axes show the flux through each enzyme's reaction for each simulation as a percent of the optimal steady state flux rate. The colour indicates the final biomass production rate at each flux rate coordinate on the plot, where white indicates no biomass production rate, and dark blue indicates the highest biomass production rate (legend shown on the right).

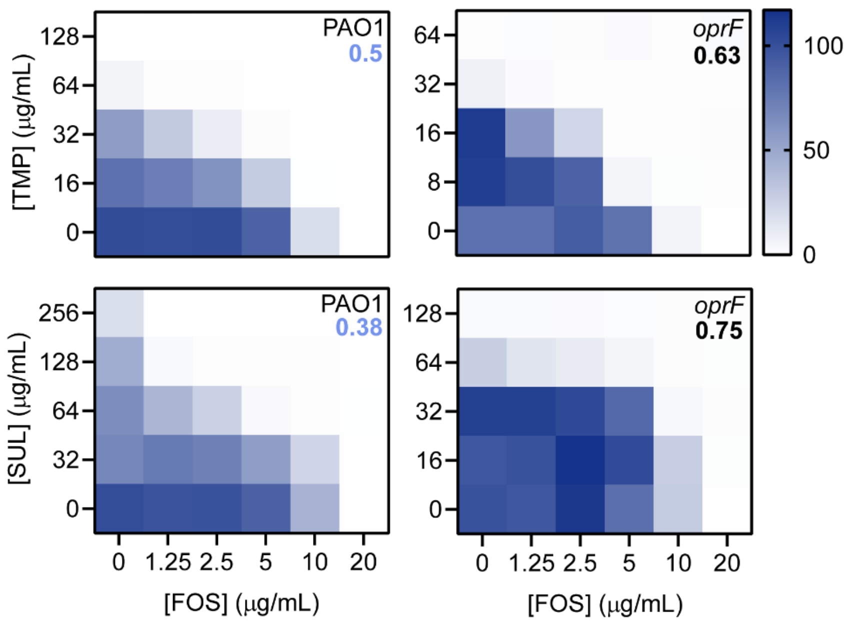

Extended Data Figure 6. Loss of OprF is redundant with FOS potentiation. Checkerboard assays were performed with an 8x8 grid and condensed to a 5x6 grid. The antibiotics used in each assay are shown on the axes, where the top two heatmaps used TMP and FOS, while the bottom two used SUL and FOS. The strain used for each assay is indicated in the top right corner. Since the *oprF* mutant is more sensitive to antifolates, a lower concentration range was used. Growth was calculated as a percent of the untreated control and was plotted as a colour gradient where white indicates no growth and dark blue indicates the highest growth (legend shown in the top right). The FICIs for TMP-FOS are 0.5 for PAO1 and 0.625 for *oprF;* and for SUL-FOS are 0.375 for PAO1 and 0.625 for *oprF*. Checkerboards were repeated in biological duplicate and a representative replicate is shown. FICIs are shown in the top right corner.

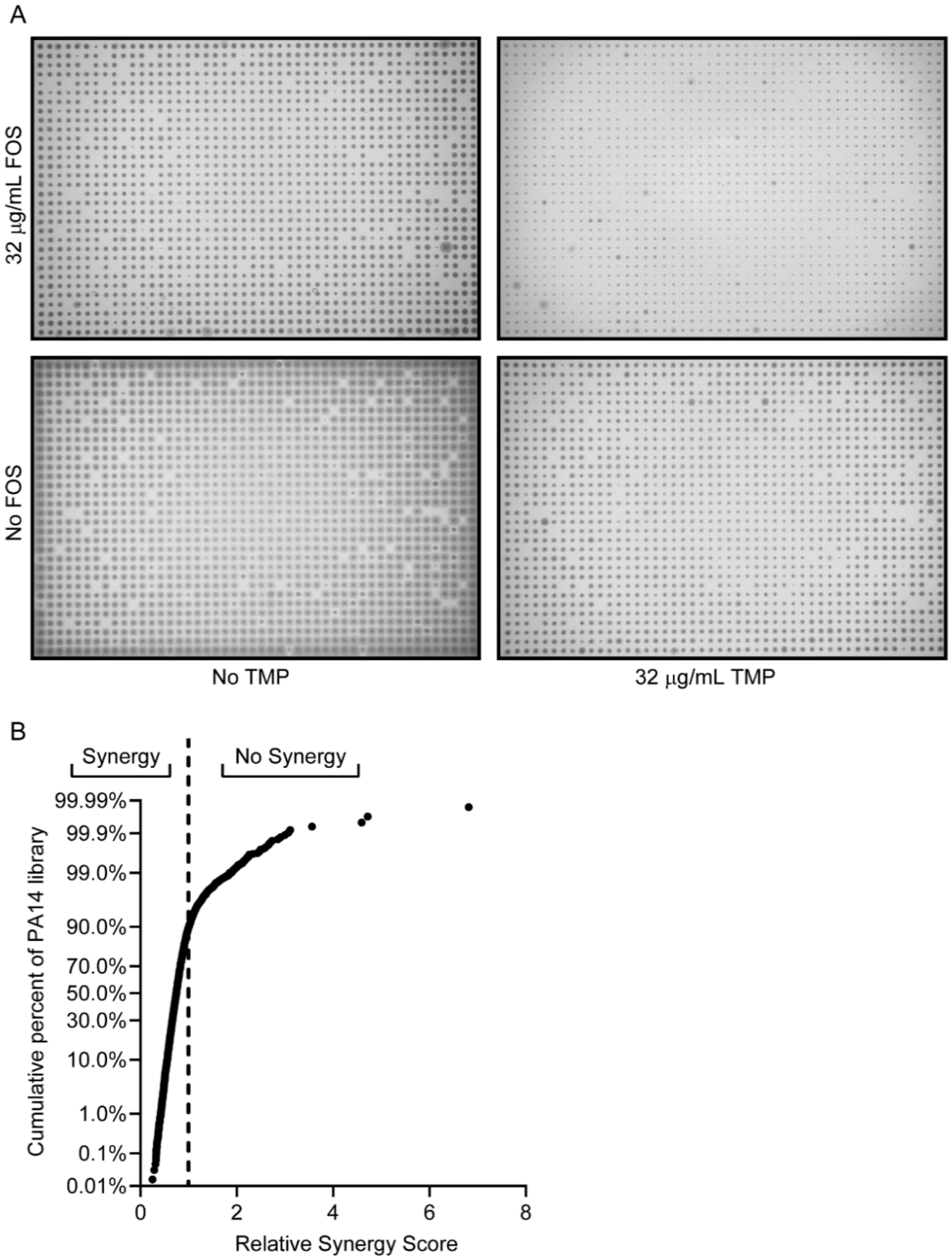

Extended Data Figure 7. A 1536-colony density chemical genetic screen identifies antibiotic interaction determinants. A) Scans of representative agar plates with 1536 colonies, where each colony is a PA14 transposon mutant (one quarter of the library is shown). The top two plates contain ¼ MIC FOS and the right two plates contain ¼ MIC TMP. **B)** A dot plot showing the distribution of synergy across the PA14 transposon library. The cumulative percentage of the library at or below a given synergy score is plotted on the Y-axis, and synergy score on the X-axis. The dashed line indicates the cutoff for synergy. Synergy occurs in over 90% of the mutants. Note that mutants hypersensitive to a single antibiotic are likely to score as lacking synergy, increasing the frequency of non-synergistic mutants.

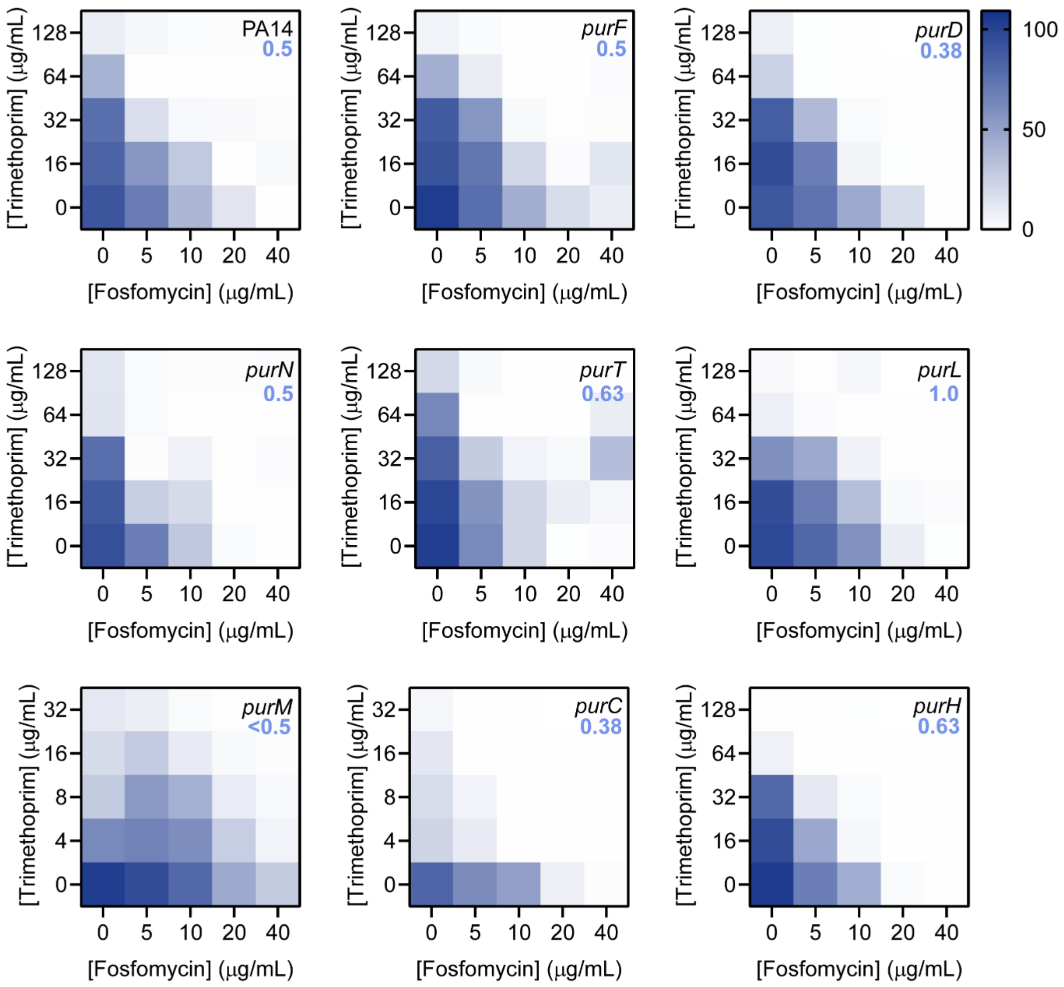

Extended Data Figure 8. Effects of disrupting purine biosynthesis on the TMP FOS interaction. Heatmaps showing 5x5 checkerboards that condense data from 8x8 checkerboards. The intensity of the blue colour corresponds to the growth as a percent of the vehicle control shown in the legend beside the top right heatmap. The strain used in each assay is indicated in the top right corner. The correct transposon insertion for each *pur* mutant was validated by colony PCR. Each checkerboard was repeated in biological triplicate, and the mean growth at every matched antibiotic combination was plotted. FICI values are shown in the top right corner.

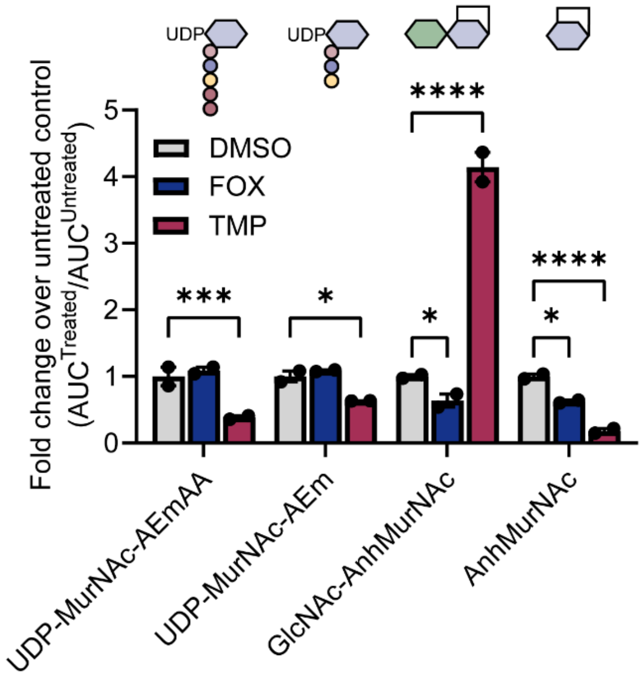

Extended Data Figure 9. TMP treatment leads to accumulation of the GlcNAc-anhMurNAc PG recycling intermediate. The abundance of soluble PG metabolites was measured by LC-MS and quantified by integrating the peak of the extracted ion chromatogram corresponding to each species' M/Z. The PG metabolites are listed on the X-axis and a cartoon of each is shown above the corresponding bar. AEmAA = the five amino acids of the stem peptide. The Y-axis shows the integrated peak value or area under the curve (AUC) for each condition and species divided by AUC of the DMSO control sample for the matched species, and the data are represented as the fold-change relative to the control. Therefore, each DMSO condition has a mean fold change of 1. Two biological replicates were performed, each with two technical replicates. One representative biological replicate is shown, and each individual data point is shown as a black circle. The bars indicate the mean of the technical replicates, and the error bars show the standard of the mean. The grey, blue, and red bars correspond to DMSO, FOX (50 µg/mL), and TMP (64 µg/mL) treated samples, respectively. A two-way ANOVA followed by Dunnett's multiple comparisons test was performed to compare the FOX and TMP treated conditions to the DMSO control. *=p<0.05, ***=p<0.001, ****=p<0.0001.

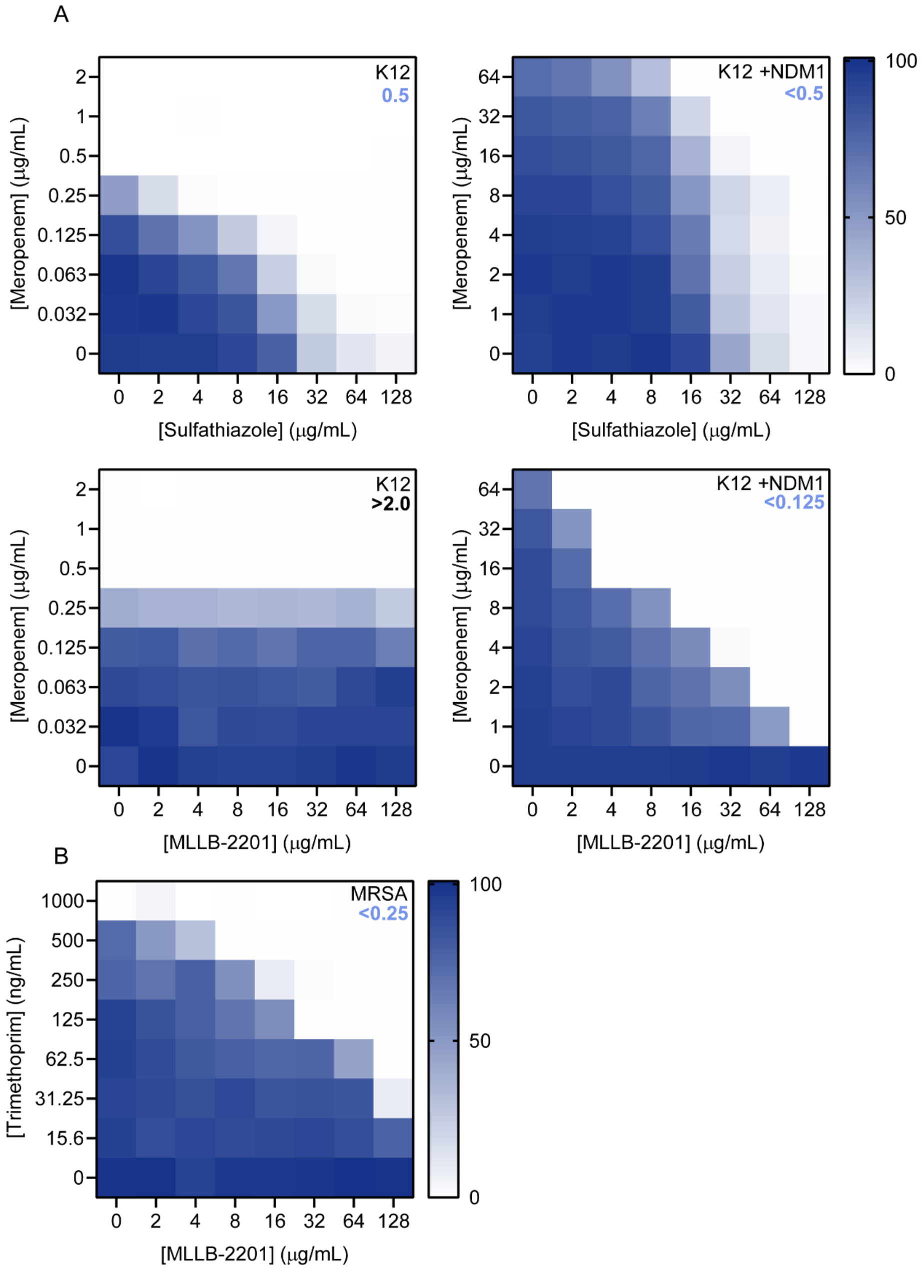

Extended Data Figure 10. MLLB-2201 synergizes with meropenem and trimethoprim. A) Checkerboard assays with *E. coli* K12 lacking NDM-1 (left) or expressing NDM-1 (right). The top heatmaps show meropenem in combination with sulfathiazole and the bottom heatmaps show meropenem in combination with MLLB-2201. **B)** A checkerboard assay against *S. aureus* USA300 (methicillin-resistant) showing synergy between trimethoprim and MLLB-2201. All checkerboard assays were performed in biological duplicate.
