# Supplementary material for: Perturbation of Pseudomonas aeruginosa peptidoglycan recycling by anti-folates and design of a dual-action inhibitor
Source: mBio. 2025 Jan 29;16(3):e02984-24. doi: 10.1128/mbio.02984-24 (PMC11898565; doi:10.1128/mbio.02984-24)

Supplementary information for:

Perturbation of *Pseudomonas aeruginosa* peptidoglycan recycling by anti-folates and design of a dual-action inhibitor

Luke N. Yaeger[1], David Sychantha[1], Princeton Luong[1], Shahrokh Shekarriz[1], Océane Goncalves[2], Annamaria Dobrin[1], Michael R. Ranieri[1], Ryan P. Lamers[1], Hanjeong Harvey[1], George C. diCenzo[3], Michael Surette[1], Jean-Philippe Côté[2], Jakob Magolan[1], and Lori L. Burrows[1*]

[1]Department of Biochemistry and Biomedical Sciences, and the Michael G. DeGroote Institute for Infectious Disease Research, McMaster University, Hamilton, Ontario, Canada

[2]Département de Biologie, Université de Sherbrooke, Sherbrooke, Québec, Canada

[3]Department of Biology, Queen's University, Kingston, Ontario, Canada

This file contains:

Legends to Supplementary Tables S1 and S2

Experimental Procedures for the synthesis of MLLB-2201

Legends:

Supplementary Table S1. Strains, plasmids, primers, and gBlocks

The bacterial strains, plasmids, PCR primers, and gBlocks used in this study are listed.

Supplementary Table S2. Results of the sub-MIC FOS-TMP synergy screen.

The first tab lists all data from the PA14 transposon mutant library synergy screen using 4 different growth conditions (no antibiotic, 32 micrograms per ml fosfomycin, 32 micrograms per ml trimethoprim, or a combination of both antibiotics at 32 micrograms per ml each). Hits were defined as actual (observed) growth divided by expected growth (based on single antibiotic plates) = greater than 1, and are shown on a separate tab. Hits that were 2 and 3 standard deviations from the mean are shown on additional tabs. The first 4 column labels Index, PlateIndex, AssayPlate, Label refer to the position of an individual mutant in the source plates. PA14 Gene.Locus, PAO1.ortholog, Gene.Name, Gene.Description provide PA14 and PAO1 ortholog gene identifiers and putative function, where available. The no drug, FOS 32, TMP 32, Combo 32 columns list the integrated colony growth, derived as described in the Methods. The TMP/no drug, FOS/no drug, Expected Combo Growth, actual Combo growth, and =actual/expected columns provide the actual and/or estimated ratios of the integrated growth for each of the relevant conditions.

Synthetic Experimental Procedures:

General:

Chemical shifts in ^{1}H NMR are reported in parts per million (ppm) relative to tetramethylsilane (TMS), with calibration of the residual solvent peaks according to values reported by Gottlieb et al. (chloroform: δ_H 7.26; DMSO: δ_H 2.50).[1] When peak multiplicities are given, the following abbreviations are used: s, singlet; d, doublet; t, triplet; q, quartet; sept., septet; dd, doublet of doublets; m, multiplet; br, broad; app., apparent; *gem*, geminal. ^{1}H NMR spectra were acquired at 400 or 700 MHz with a default digital resolution (Brüker parameter: FIDRES) of 0.22 and0.15 Hz/point, respectively. Coupling constants reported herein therefore have uncertainties of ±0.4 Hz and ±0.3 Hz, respectively. Exchangeable proton peaks sometimes went unobserved. Synthetic experimental procedures were adapted from work previously published by Davies et al.[1] Reactions were carried out at room temperature (rt) if temperature is not specified. Compounds purified by both normal-phase and reverse-phase flash chromatography used Teledyne Combi*Flash* Rf+ and NextGen 300+ purification systems (www.teledyneisco.com) equipped with pre-packed silica cartridges (either 40–60 μM or 20–40 μM particle size). Low-resolution mass spectral (LRMS) measurements were recorded on an Advion Expression CMS Compact Mass Spectrometer (Albany, NY). High-resolution mass spectrometric (HRMS) data was obtained using a Brüker micrOTOF II system with electrospray ionization (ESI) and paired with an Agilent HPLC and UV detector.

Reagents were purchased from Ambeed, Combi-Blocks, Fisher Scientific, and Sigma Aldrich, and used without any additional purification.

[1] Davies, D.T.; Leiris, S.; Sprynski, N.; Castandet, J.; Lozano, C.; Bousquet, J.; Zalacain, M.; Vasa, S.; Dasari, P.K.; Pattipati, R.; Vempala, N.; Gujjewar, S.; Godi, S.; Jallala, R.; Sathyap, R.R.; Darshanoju, N.A.; Ravu, V.R.; Juventhala, R.R.; Pottabathini, N.; Sharma, S.; Pothukanuri, S.; Holden, K.; Warn, P.; Marcoccia, F.; Benvenuti, M.; Pozzi, C.; Mangani, S.; Docquier, J.D.; Lemonnier, M.; and Everett, M. ANT2681: SAR Studies Leading to the Identification of a Metallo-β-lactamase Inhibitor with Potential for Clinical Use in Combination with Meropenem for the Treatment of Infections Caused by NDM-Producing Enterobacteriaceae. *ACS Infectious Diseases*, **2020**, *6*, 2419-2430.

Ethyl 5-((4-nitrophenyl)sulfonamido)thiazole-4-carboxylate

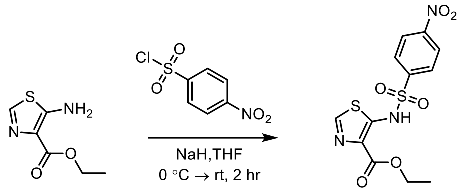

To a clean, dry 50 mL round bottom flask, sodium hydride (60% in oil, 66.9 mg, 1.74 mmol, 3 equiv.) was suspended in dry tetrahydrofuran (THF). The mixture was cooled to 0 °C in an ice bath before the addition of ethyl 5-amino-thiazole-4-carboxylate (100.0 mg, 0.58 mmol, 1 equiv.) and stirred for at least ten minutes. Afterward, 4-nitrobenzenesulfonyl chloride (154.4 mg, 0.69 mmol, 1.2 equiv.) was added at 0 °C and the mixture was left to warm to room temperature and stir for 2 hours. Reaction progress was monitored with thin layer chromatography (TLC) [5% methanol (MeOH)/dichloromethane (DCM)]. The reaction was quenched with an aqueous solution of saturated ammonium chloride and diluted with diethyl ether. The resulting precipitate was then collected via vacuum filtration and washed with multiple aliquots of diethyl ether and water. The product was dried under vacuum to obtain a brownish-yellow solid. The crude product was purified using normal-phase chromatography on silica gel (0→20% MeOH/DCM) to afford the sulfonamide as an orange-yellow solid (114.0 mg, 0.31 mmol, 55 %). If desired, the crude product could be carried forward to the next reaction without any prior purification.

R_f = 0.34 (5% MeOH/DCM).

^{1}H NMR (400 MHz, DMSO-d_6) δ 8.73 (s, 1H), 8.40 (d, J = 8.9 Hz, 2H), 8.04 (d, J = 8.9 Hz, 2H), 4.15 (q, J = 7.1 Hz, 2H), 1.17 (t, J = 7.1 Hz, 3H).

LRMS m/z: $[M + H]^+$ calculated for $C_{12}H_{12}N_3O_6S_2^+$ 358.0162 ; Found 358.1500.

HRMS (ESI) m/z: $[M - H]^-$ calculated for $C_{12}H_{10}N_3O_6S_2^-$ 356.0017; Found 356.0020.

Ethyl 5-((4-aminophenyl)sulfonamido)thiazole-4-carboxylate

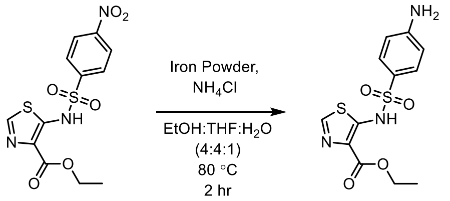

In a clean, dry 50 mL round bottom flask, Ethyl 5-((4-nitrophenyl)sulfonamido)thiazole-4 carboxylate (100.0 mg, 0.28 mmol, 1 equiv.) was dissolved in a mixture of ethanol, THF, and water (4:4:1), capped with a reflux condenser, and placed under an argon atmosphere. Iron powder (78.1 mg, 1.40 mmol, 5 equiv.) and ammonium chloride (37.4 mg, 0.70 mmol, 2.5 equiv.) were added to the reaction vessel at room temperature before heating to 80 °C and stirring for at least 1 hour. Reaction progress was monitored with TLC (5% MeOH/DCM). Upon consumption of all the starting material, the reaction mixture was filtered hot, and the leftover residue was rinsed with a solution of 10% MeOH in DCM. The resulting filtrate was washed with water, dried over sodium sulphate (Na_2SO_4), filtered, and concentrated under reduced pressure. The product was purified using normal-phase chromatography on silica gel

(0→20% MeOH/DCM) to provide a yellow-brown oil, which upon lyophilization, became pale yellow/ off-white powder (61.4 mg, 0.19 mmol, 67%).

R_f = 0.53 (5% MeOH/DCM).

^{1}H NMR (400 MHz, DMSO-d_6) δ 8.61 (s, 1H), 7.47 (d, J = 8.8 Hz, 2H), 6.58 (d, J = 8.8 Hz, 2H), 4.24 (q, J = 7.1 Hz, 2H), 1.25 (t, J = 7.1 Hz, 3H).

LRMS m/z: $[M + H]^+$ calculated for $C_{12}H_{14}N_3O_4S_2^+$ 328.0420 ; Found 328.2000.

HRMS (ESI) m/z: $[M - H]^-$ calculated for $C_{12}H_{12}N_3O_4S_2^-$ 326.0275; Found 326.0283.

5-((4-aminophenyl)sulfonamido)thiazole-4-carboxylic acid (MLJB-2201)

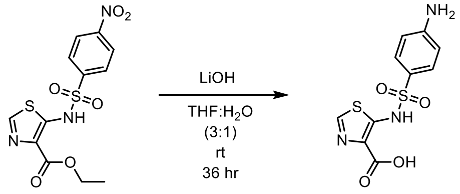

MLLB-2201

Ethyl 5-((4-aminophenyl)sulfonamido)thiazole-4-carboxylate (90 mg, 0.27 mmol, 1 equiv.) was dissolved in a 3:1 THF:H_2O mixture. To this, lithium hydroxide monohydrate (26.34 mg, 1.10 mmol, 4 equiv.) was added and the mixture was left to stir for ~36 hours. The reaction mixture was diluted with water and washed with ethyl acetate to remove any residual starting material. The aqueous layer was cooled in an ice bath and carefully acidified with 1M hydrochloric acid to approximately pH 4, lest the free amine become protonated, and the product remain in the aqueous phase. Literature described the product precipitating out upon acidification, but this approach was found to be ineffective in readily isolating the target in our hands. Instead, the aqueous layer was then extracted with DCM and location of the product was monitored using TLC (5% MeOH/DCM). The product remained largely baseline on regular phase silica plates up to 20% MeOH/DCM, but the product moved from the aqueous to the organic phase provided that the species retained a net neutral charge. Should protonation of the free amine occur, and the product remain in the aqueous phase, the pH could be adjusted using mild base (e.g., aqueous sodium bicarbonate) and the mixture was extracted with additional DCM. The solvent of the organic phase was allowed to evapourate sitting out at room temperature to obtain an off-white solid. Note that the product was found to be very heat sensitive and capable of readily undergoing decarboxylation even upon mild heating on a rotovap. The crude product was purified using reverse-phase chromatography on C18 silica gel (10→100% ACN/H_2O). The collected fractions were freeze-dried to afford the product, MLLB-2201 as an off- white solid (12.3 mg, 0.04mmol, 15%)

^{1}H NMR (400 MHz, DMSO-d_6) δ 7.97 (s, 1H), 7.35 (d, J = 8.5 Hz, 2H), 6.49 (d, J = 8.6 Hz, 2H), 5.65 (s, 2H).

HRMS (ESI) m/z: $[M + H]^+$ calculated for $C_{12}H_{10}N_3O_4S_2^+$ 300.0107; Found 300.0104.

^{1}H NMR Spectra

Ethyl 5-((4-nitrophenyl)sulfonamido)thiazole-4-carboxylate (^{1}H NMR; 400 MHz; DMSO-d_6)

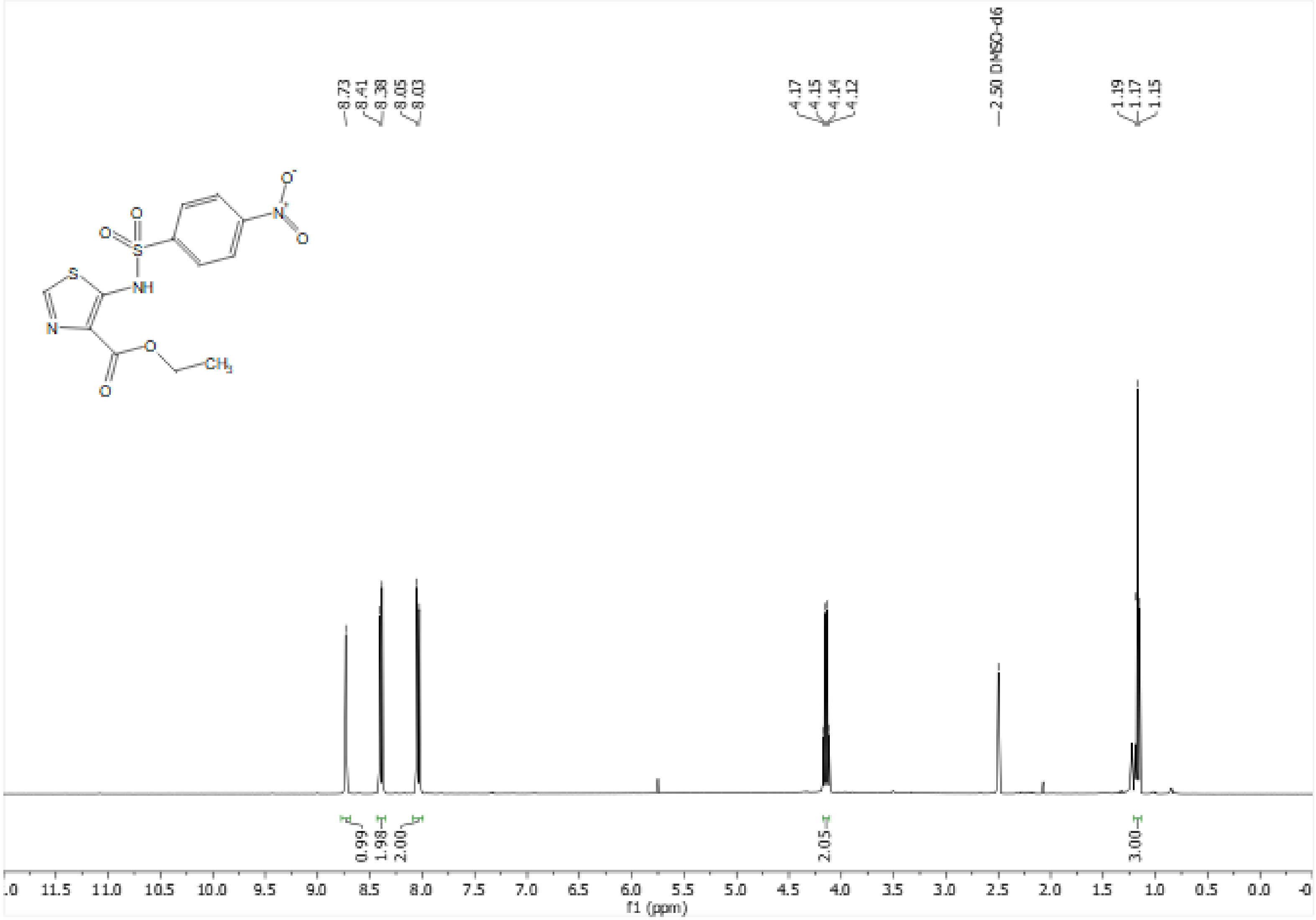

Ethyl 5-((4-aminophenyl)sulfonamido)thiazole-4-carboxylate (^{1}H NMR; 400 MHz; DMSO-d_6)

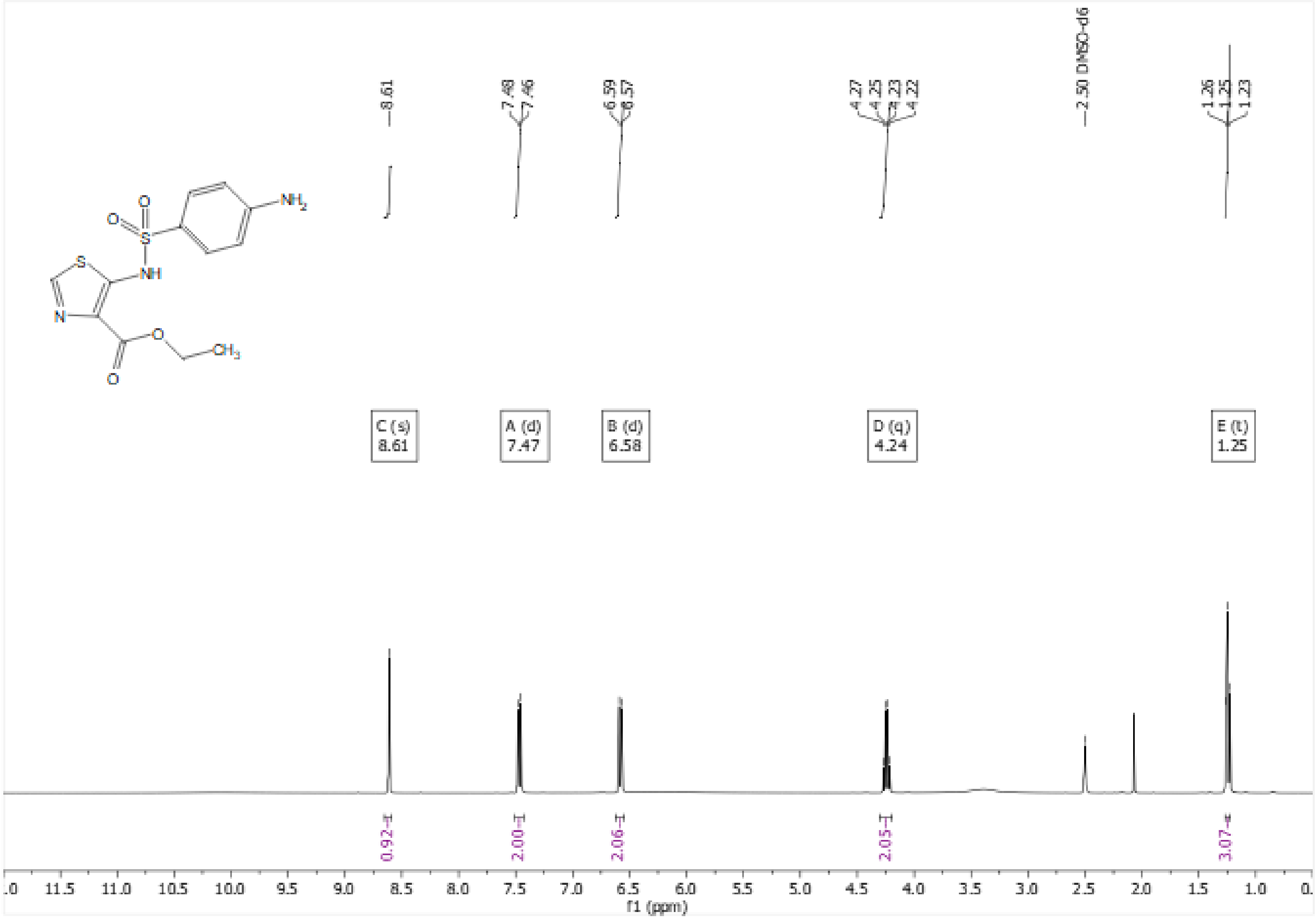

5-((4-aminophenyl)sulfonamido)thiazole-4-carboxylic acid (MLJB-2201) (^{1}H NMR; 400 MHz; DMSO-d_6)

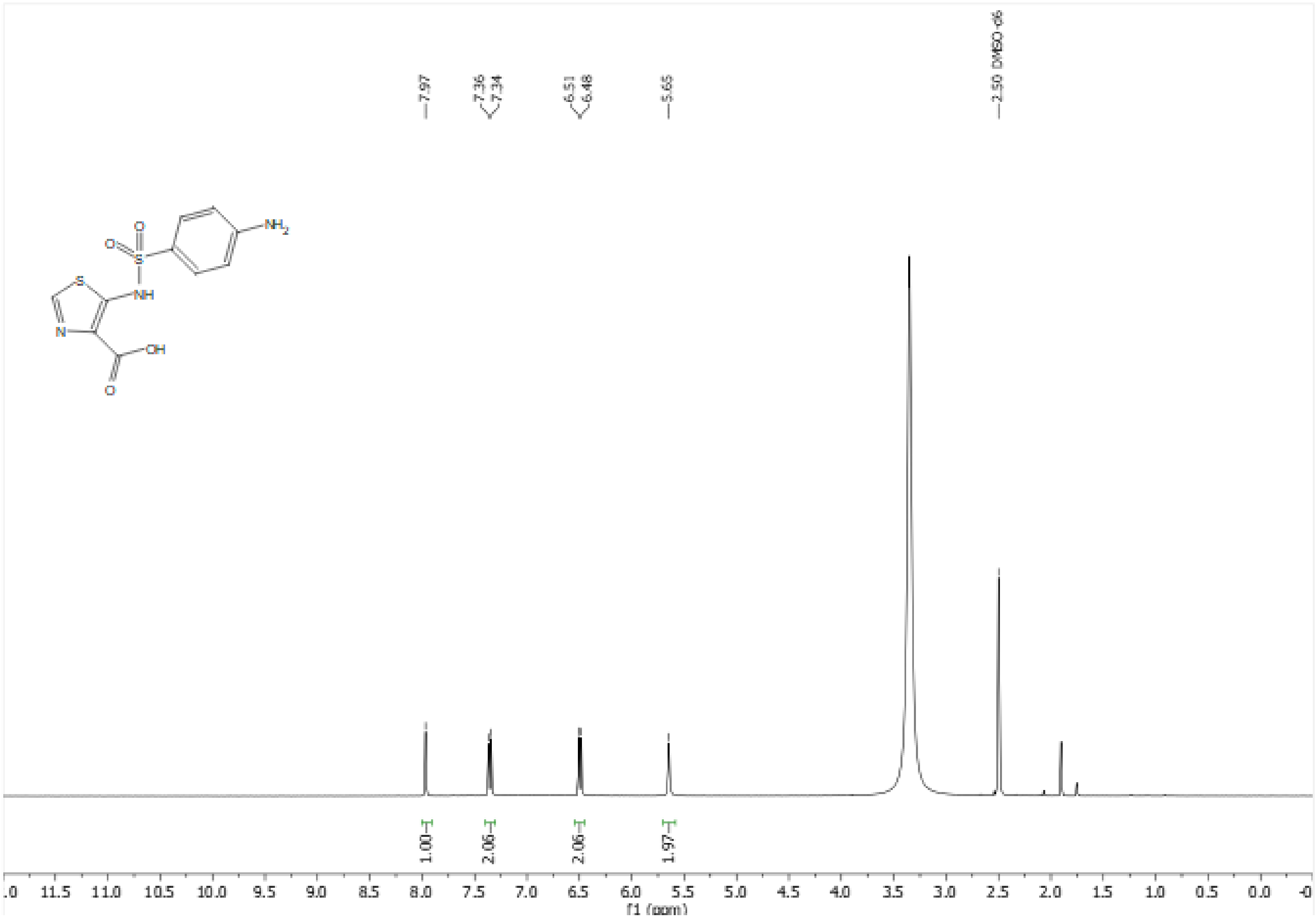

Supplement: Supplemental Information — Captions to Tables S1 and S2, plus the methods and references for synthesis of MLLB-2201 and validation of the molecule's structure by NMR. [file mbio.02984-24-s0002.pdf]
