# Supplementary material for: Perturbation of Pseudomonas aeruginosa peptidoglycan recycling by anti-folates and design of a dual-action inhibitor
Source: mBio. 2025 Jan 29;16(3):e02984-24. doi: 10.1128/mbio.02984-24 (PMC11898565; doi:10.1128/mbio.02984-24)
Supplement: Table S1 — Strains and primers used for this study. [file mbio.02984-24-s0003.docx]

**Supplementary Table S1. Strains, plasmids, primers, and gBlocks**

| **Strains** | | |
| --- | --- | --- |
| **Name** | **Description** | **Source** |
| PAO1 | Wild-type *P. aeruginosa* strain graciously donated by Keith Poole (Queen's University, Kingston, Canada) | ^1^ |
| *ampC* | An in-frame deletion mutant of *ampC* in PAO1 | This work |
| *ampR* | An in-frame deletion mutant of *ampR* in PAO1 | This work |
| *dacBC*** | A PAO1 mutant containing DacB S72A and DacC S64A catalytically inactive point mutations | This work |
| *dacBC**ampG* | A PAO1 mutant containing DacB S72A and DacC S64A point mutations and an in-frame deletion mutant of *ampG* | This work |
| *ampC* S90A | A PAO1 mutant containing an AmpC S90A catalytically inactive point mutation | This work |
| *oprF* | An *oprF* FRT mutant with the Gentamicin cassette flipped out | ^2^ |
| PAO1 +pUCP20 (Empty) | Wild-type PAO1 containing the pUCP20 empty plasmid. Carbenicillin resistant | This work |
| PAO1 +pUCP20 (*DHFRII*) | Wild-type PAO1 containing the pUCP20 plasmid expressing the *DHFRII* gene cloned from the pMS402 backbone. Carbenicillin resistant | This work |
| PA14 | Wild-type *P. aeruginosa* strain | ^3^ |
| *purC* | A PA14 mutant containing a transposon insertion in *purC*. Gentamicin resistant | ^3^ |
| *purD* | A PA14 mutant containing a transposon insertion in *purD*. Gentamicin resistant | ^3^ |
| *purF* | A PA14 mutant containing a transposon insertion in *purF*. Gentamicin resistant | ^3^ |
| *purH* | A PA14 mutant containing a transposon insertion in *purH*. Gentamicin resistant | ^3^ |
| *purL* | A PA14 mutant containing a transposon insertion in *purL*. Gentamicin resistant | ^3^ |
| *purM* | A PA14 mutant containing a transposon insertion in *purM*. Gentamicin resistant | ^3^ |
| *purN* | A PA14 mutant containing a transposon insertion in *purN*. Gentamicin resistant | ^3^ |
| *purT* | A PA14 mutant containing a transposon insertion in *purT*. Gentamicin resistant | ^3^ |
| *glpT* | A PA14 mutant containing a transposon insertion in *glpT*. Gentamicin resistant | ^3^ |
| *E. coli* K12 BW25113 | A wild-type *E. coli* strain with the genotype [F−Δ(*araD-araB*)567 *lacZ*4787Δ::*rrnB*-3 LAM− *rph*-1 Δ(*rhaD-rhaB*)568 *hsdR*514] | ^4^ |
| K12 + NDM1 | *E. coli* K12 BW25113 containing the pGDW plasmid expressing NDM-1. Kanamycin resistant | ^5^ |
| *E. coli* DH5α | A wild-type *E. coli* strain with the genotype F– *endA1 gln*V44 *thi*-1 *recA1 relA1 gyrA96 deoR nupG purB20 φ80dlacZΔM15 Δ(lacZYA-argF)U169*, *hsdR17(rK–mK+)*, λ– | Invitrogen |
| *E. coli* SM10 | A wild-type *E. coli* strain used for efficient conjugative transfer of plasmid DNA to *P. aeruginosa* | ^6^ |
| *S. aureus* USA300 | A wild-type *S. aureus* strain. Methicillin resistant | ^7^ |
| **Plasmids** | | |
| **Name** | **Description** | **Source** |
| pMS402 (Empty) | A plasmid containing a promoter-less *luxBCDE* cassette. Kanamycin and trimethoprim resistant | ^8^ |
| pMS402Gm (Empty) | A plasmid containing a promoter-less *luxBCDE* cassette. Gentamicin and trimethoprim resistant | This work |
| pMS403 (Empty) | A plasmid containing a promoter-less *luxBCDE* cassette. Gentamicin resistant | This work |
| pMS403 (P*ampC*) | A plasmid containing the *luxBCDE* cassette under control of the *ampC* promoter. Gentamicin resistant | This work |
| pGDW (*NDM1*) | A plasmid expressing NDM-1. Kanamycin resistant | ^5^ |
| pUCP20 (Empty) | A plasmid with expression controlled by a *lac* promoter. Ampicillin/carbenicillin resistant | ^9^ |
| pUCP20 (*DHFRII*) | A plasmid with the *DHFRII* (*folA*) gene from pMS402 under control of a *lac* promoter. Ampicillin/carbenicillin resistant | This work |
| pEX18Gm (Empty) | A plasmid for performing allelic exchange. Gentamicin resistant | ^10^ |
| pEX18Gm (*ampC*) | The pEX18Gm plasmid containing the *ampC* deletion construct gblock. Gentamicin resistant | This work |
| pEX18Gm (*ampC* S90A) | The pEX18Gm plasmid containing the *ampC* S90A point mutation gblock. Gentamicin resistant | This work |
| pEX18Gm (*ampG*) | The pEX18Gm plasmid containing the *ampG* deletion construct gblock. Gentamicin resistant | This work |
| pEX18Gm (*ampR*) | The pEX18Gm plasmid containing the *ampR* deletion construct | This work |
| pUC57 (*ampC*) | The pUC57 plasmid containing the *ampC* deletion construct gblock. Ampicillin resistant | Genscript |
| pUC57 (*ampC* S90A) | The pUC57 plasmid containing the *ampC* S90A point mutation gblock. Ampicillin resistant | Genscript |
| pUC57 (*ampG*) | The pUC57 plasmid containing the *ampG* deletion construct gblock. Ampicillin resistant | Genscript |
| pPS856 | A plasmid containing a gentamicin resistance cassette within a multiple cloning site. Used to construct pMS402Gm | ^10^ |
| **Primers** | | |
| **Name** | **Sequence (5’-3’)** | |
| P*ampC* Fwd | ATATGGATTCAGGGCGTTCAGCGGCAAATGG | |
| P*ampC* Rvs | TTAAGGATCCATTGGCGTCCTTTGTCGTTGGCTGC | |
| d*ampR* Up Fwd | TATAAAGCTTTCCGCCGTTTCGCCGCAATCTCC | |
| d*ampR* Up Rvs | GAAGCTTCGAAGGCGCGCAGGGCGTTCAGCGGCAAATGGGGTCGAACC | |
| d*ampR* Dwn Fwd | AACGCCCTGCGCGCCTTCGAAGCTTCCGGTTATGCAGGCGATTCAAGTGTCG | |
| d*ampR* Dwn Rvs | AATTGGATCCGCTGGCTGGCGCGGTCGTCG | |
| *DHFRII* Fwd | ATCAGAATTCGATTCACAAGAAGGATTCGACATGGG | |
| *DHFRII* Rvs | ACTCAAGCTTCGTAAGATGCTTTTCTGTGACTGG | |
| *purFtn Fwd* | ATCTTTCCAGCTTGAGCCAAGTAGGGG | |
| *purFtn Rvs* | CCGCTCTCCCCTTTGTCAGTCG | |
| *purDtn Fwd* | CGCCAGGAGAACCCCATGAACG | |
| *purDtn Rvs* | AAGTCCCTTCGAAGGTGAATGGCCG | |
| *purNtn Fwd* | CGTGGTCCTGAACAATCTGAAAAACC | |
| *purNtn Rvs* | CATCTGTTTCCAGTCGGCGGTATAGC | |
| *purTtn Fwd* | GGATTCCGTTTTCTTTCATTTCCGAGGTTCTTCC | |
| *purTtn Rvs* | CGGCGCATCTCAGAGTTCGACG | |
| *purLtn Fwd* | TTCCCGTCTCCAGAGGCTGTTCCG | |
| *purLtn Rvs* | CGGCGAACCTCGGGAAGAAGGTCG | |
| *purMtn Fwd* | GATCTCCGAATTACCCCTATAGGCCTGG | |
| *purMtn Rvs* | TCAGCACCACGACATTGCAGAGTTTCG | |
| *purCtn Fwd* | AGGCCCTATTACCCGTAAGCGGAGC | |
| *purCtn Rvs* | TTCTTTCAGATGGTTGCGCGCGTTAGG | |
| *purHtn Fwd* | CCAGCCCTCAGGACCCTTGC | |
| *purHtn Rvs* | GCGTTATCCGCCCTGCGTGAAATTCG | |
| **gBlocks** | | |
| **Name** | **Sequence (5’-3’)** | |
| *ampC* deletion construct | gaattcTCAGCCAGTAGCTGCCGGTACTCACTTCGGTGGCGAACGGACGCCGGATGCTCTCGCTGGCCAGTTGCCGGGCAAACATCGCCGCCGGCGCCAGGGCCACGCCGACACCCTGGCGGGCCGCCTCGAGCATGGCCAGCGAGGTGTCGAAGACGATGCTCCGGGTCAGTGGCGCGTGCGCCGGCAGTCCGGCCGCCTGGAACCACAGCGGCCACTCGTCGGCGCGGTAGGAGCGCAGCAGGGTGTGCTGCAGCAGGTCGGCGGGACTGTGCAACTGGGCGGCGACCTCCGGGCAGCAGAGCACCGTCAGCGGCGCCTCGAACAACGCCAGCGCCTCGGTGCCGTGCCAGGCGCCGCCGCCGAAGCGGATCGCGTAGTCGAGCCCCTCGGCGGCGATGTCGACGCGGTTGTTGTGGGTGGACAGGCGCAGATCGATGAAGGGATGGCGCGCCTGGAAGTCCTCCAGCCGCGGCAGCAGCCAACCGACCGTGAAGGTTCCGACCGCGCCGACGGTGAGCACGTCCCGGTAGTGGCCACCCTCGAAACGTTCCAGCAGGCCGGCGATGCGGTCGAAGGAGTCACACAGCACCGGCAGCAGGCTCTCGCCCTCGTGGGTCAGCATGAGGCCGCGCGGCAGACGCTTGAACAGGGCCACGCCGAGACGCTCCTCGAGGCTCTTCACCTGGTGGCTGACCGCCGCCTGGGTCACGCACAGCTCGATGGCCGCGCGGGTGAAGCTCAGGTGCCGGGCCGCTCGCGAGGGCGACGGAGCGTAGCGGCGCGGGACGCCGGTCCTGGCTATGATGGTGCCATGAGCGCTTCCCCGCCCCTCCGCCAGCCCTGCCCGCCCGGCGCCTGCGTCTGCGAACGCGAGCGCCTGGAGGCGCCCGGCGCGGACCGTCGCATCCTCCTCCTGACCCGCCAGGAAGAGCAGCGCCTGGCCGCTCGCCTGGAAGCCCTGCGCAGCCTGGAAGACCTGGAACACCTGCTGCGGCGCATGGAGGAACAACTGGGCATCCGCCTGCGGATCGCCCCGGCCTTCGGCGAGGTGCGCAGCATGCGCGGCATCCGCATGCGCTTCGAGGAGCAGCCGGGGCTCTGCCGCAAGACCCGCCAGGCGATTCCCGCCGCCATCCGCCGCGGCCTGGAGAAGCGCCCGGAAGTCGCCTACGCCCTGCTCAACGCCCATGACCTGCTGCGCGACGCCTGAAGGTACTGAACGACAGGAAGAGGATGTCGCTCAAGACCTGGGCGGGCTTCAGGAGTATCGGCGGATAACGCCCATGGCGTTATTCGCCCTACAGGCCGCAGCGGATGCAGGCGAGCCCCGGGTCCGCCTGAATCCTTGGCGGGACTGGCCACCGCGCCGGGGTTCAGCTCGCCGACTCCGCCTGCACGTCGCCGAACATCGCCTGCAGGCGGAGCAGGCAGGCATCGCACAGGGTGCGCAGTTCGTCGAGGCGGATGCCGGCGAGGATTTCCATGCCGCGCAGCGGATCGGCGaagctt | |
| *ampC* S90A point mutation | gaattcATGCGCGATACCAGATTCCCCTGCCTGTGCGGCATCGCCGCTTCCACACTGCTGTTCGCCACCACCCCGGCCATTGCCGGCGAGGCCCCGGCGGATCGCCTGAAGGCACTGGTCGACGCCGCCGTACAACCGGTGATGAAGGCCAATGACATTCCGGGCCTGGCCGTAGCCATCAGCCTGAAAGGAGAACCGCATTACTTCAGCTATGGGCTGGCCTCGAAAGAGGACGGCCGCCGGGTGACGCCGGAGACCCTGTTCGAGATCGGCGCGGTGAGCAAGACCTTCACCGCCACCCTCGCCGGCTATGCCCTGACCCAGGACAAGATGCGCCTCGACGACCGCGCCAGCCAGCACTGGCCGGCACTGCAGGGCAGCCGCTTCGACGGCATCAGCCTGCTCGACCTCGCGACCTATACCGCCGGCGGCTTGCCGCTGCAGTTCCCCGACTCGGTGCAGAAGGACCAGGCACAGATCCGCGACTACTACCGCCAGTGGCAGCCGACCTACGCGCCGGGCAGCCAGCGCCTCTATTCCAACCCGAGCATCGGCCTGTTCGGCTATCTCGCCGCGCGCAGCCTGGGCCAGCCGTTCGAACGGCTCATGGAGCAGCAAGTGTTCCCGGCACTGGGCCTCGAACAGACCCACCTCGACGTGCCCGAGGCGGCGCTGGCGCAGTACGCCCAGGGCTATGGCAAGGACGACCGCCCGCTACGGGTCGGTCCCGGCCCGCTGGATGCCGAAGGCTACGGGGTGAAGACCAGCGCGGCCGACCTGCTGCGCTTCGTCGATGCCAACCTGCATCCGGAGCGCCTGGACAGGCCCTGGGCGCAGGCGCTCGATGCCACCCATCGCGGTTACTACAAGGTCGGCGACATGACCCAGGGCCTGGGCTGGGAAGCCTACGACTGGCCGATCTCCCTGAAGCGCCTGCAGGCCGGCAACTCGACGCCGATGGCGCTGCAACCGCACAGGATCGCCAGGCTGCCCGCGCCACAGGCGCTGGAGGGCCAGCGCCTGCTGAACAAGACCGGTTCCACCAACGGCTTCGGCGCCTACGTGGCGTTCGTCCCGGGCCGCGACCTGGGCCTGGTGATCCTGGCCAACCGCAACTATCCCAATGCCGAGCGGGTGAAGATCGCCTACGCCATCCTCAGCGGCCTGGAGCAGCAGGGCAAGGTGCCGCTGAAGCGCTGAaagctt | |
| *ampG* deletion construct | gagctcGTTGCAGTTCACCAACTTCCGCGATTGACGAGGTTTTTCGGAACCTCTCGCCATCCCGGGCATCGCAATAGGGAGTAGCGCCGCCGCGACCTCTCCCGCGTTGTCCGCCCCTGTCGGCGCAGCGTCGGTTCGTCCGCGCTGCGCCGATTCGTATCCAGGCCCGGAAACGTCGCCCCACGGCGTCCGGGCAGGAATCTGCAAGCCCGTCCCGTTTCCGTTGCGGAAGGGAGGGGTGTTTTTTCGAGAAATGGAGGTAATGCATGGAATTGAACTACGACCGACTGGTGCAGCAGACCGAGTCCTGGCTGCCGATCGTGCTGGAGTACAGCGGCAAGGTCGCCCTGGCGCTGCTGACCCTGGCGATCGGCTGGTGGCTGATCAACACCCTGACCGGCCGGGTCGGCGGCCTGCTCGCCAGGCGCAGCGTCGACCGCACCCTGCAAGGCTTCGTCGGCAGCCTGGTGAGCATCGTCCTGAAGATCCTGCTGGTGGTCAGCGTGGCTTCCATGATCGGCATCCAGACCACCAGCTTCGTCGCCGCCATCGGCGCCGCCGGCCTGGCCATCGGCCTGGCCCTGCAGGGCAGCCTGGCTAACTTCGCCGGCGGCGTGCTGATCCTGCTGTTCCGCCCGTTCAAGGTCGGCGACTGGATCGAGGCACAGGGCGTGGCCGGCACCGTGGATTCGATCCTGATCTTCCACACCGTGCTGCGTAGCGGCGACAACAAGCGGATCATCGTGCCCAACGGGGCGCTGTCCAACGGAACGGTGACCAACTACTCCGCCGAGCCGCTGCGCAAGGTGGTCTTCGACGTCGGCATCGACTACGACGCCGATCTGAAGAATGCGCAGAACATTCTCCTGGCCATGGCCGACGATCCGCGGGTTCTGAAGGACCCGGCACCGGTGGCGGTGGTTTCCAATCTCGGCGAAAGCGCGATTACCCTGTCCCTGCGGGTCTGGGTGAAGAACGCCGACTACTGGGACGTGATGTTCATGTTCAACGAAAAGGCCCGCGACGCGCTGGGCAAGGAAGGTATCGGCATTCCCTTCCCGCAGCGGGTGGTCAAGGTTGTGCAGGGCGCGATGGCCGACTGAGGTCCGTGCGATCCACGAAAAAGGCCGGCGAATGCCGGCCTTTTTCATTCTCGCCTCTAGACGCAAAAAATAACGCGCACTCTAACCGCTCTACTTCGCTGTAAGCCAGCCATGCCGACGTATGTCCACCCTGTCGTTGTGCAGGAGAACGCCTTCCTCGCGCAAGCGTGCGCGTTGTTCGCGACCGCCGGGACTGTCCGCCGGCAGGCTGATGCGACCGCCGGCGCCGAGAACCCGATGCCAGGGCAGGCGGGTATCCACCGGCAACTGGCTGAGCGTCCTGCCGACCCAGCGGGCGGCGCGGCCTAGGCCGGCCAGTTCGGCCAGTTGCCCGTAGCTGACCACCTGCCCCGGCGGCACCTGCGCCAGCACCAGGTACAGGGCCTCGCGGCGCGCCTGGGCACTTGCCGGGTCGCTGCCGGCCCATGCATGCTCATCGGTTTTCCCTTTCCTGTCCGCCTTGTGAACGCCCATGTCGCTCAGAGCCCTCTGTTCAATCGTCGTGATGCTCCTGGCCGGCCCCGCGCTGGCCGACACCGTATGGCTGGACAACGGCGACCGCCTGTCCGGCGAGATCGTGCTGATGGACGGCGGCAAGCTGGCGCTGAAGACCCGTTATGCCGGCCAGGTGCTGATCGATTGGAAGGATATCGACACCATCAGTTCCGACAAACCCCTGCTGATCAAGCAGCAGGGCGTATCCGGGCAGCGTAGCCGAACCCTGGAGGCGGCGGGCAAGGGCATGGTGCGGATCGTCGATGGCGGCAGCCATACCGTCCCGCTGGCCAGTATTCGCCAGATGGTGCCGCCGCGACCGCTGGTGGAGGACCTGGTCTGGGAGGGCAATCTCGACGTCAAGCTGGATAGCAAGCGCAACGACAGCGACAAGGACGAATGGAAGCTCAAGGGCGATACCCGGCTTCGCCACGGTGCCTGGCGCCACGTGCTGGCGGGGGAAGTGGAGCGGGAAAAGAAGGACGGGCGCAAGGTCGAGGACAACTGGGAGCTGGACTACGACCTCGATCGCTTCTTCGACGAACACTGGTTCTGGCGCGGCAGCTATTCGCAGAAGCACGATGCGATCGACAACCTGGAGCGCCAGAGCGCGCTGGGAACCGGCCCCGGCTACCAGTTCTGGGACGACGAACTCGGGCGTTTCGACCTGGTCGCCGAGATCAGTCGCTGGCAACTGGAGTGGCGaagctt | |

**References**

1. Masuda N. Ohya S. Cross-resistance to meropenem, cephems, and quinolones in *Pseudomonas aeruginosa*. *Antimicrob. Agents Chemother.* **36**, 1847–1851 (1992).

2. Yaeger, L. N. *et al.* A genetic screen identifies a role for *oprF* in *Pseudomonas aeruginosa* biofilm stimulation by subinhibitory antibiotics. *bioRxiv* (2023).

3. Liberati, N. T. *et al.* An ordered, nonredundant library of *Pseudomonas aeruginosa* strain PA14 transposon insertion mutants. *Proc. Natl. Acad. Sci. U. S. A.* **103**, 2833–2838 (2006).

4. Baba, T. *et al.* Construction of *Escherichia coli* K-12 in-frame, single-gene knockout mutants: The Keio collection. *Mol. Syst. Biol.* **2**, 2006.0008 (2006).

5. Cox, G. *et al.* A Common Platform for Antibiotic Dereplication and Adjuvant Discovery. *Cell Chem. Biol.* **24**, 98–109 (2017).

6. Simon, R., Priefer, U. & Pühler, A. A broad host range mobilization system for *in vivo* genetic engineering: Transposon mutagenesis in Gram-negative bacteria. *Nat. Biotechnol.* **1**, 784–791 (1983).

7. Voyich, J. M. *et al.* Insights into Mechanisms Used by *Staphylococcus aureus* to Avoid Destruction by Human Neutrophils. *J. Immunol.* **175**, 3907–3919 (2005).

8. Duan, K., Dammel, C., Stein, J., Rabin, H. & Surette, M. G. Modulation of *Pseudomonas aeruginosa* gene expression by host microflora through interspecies communication. *Mol. Microbiol.* **50**, 1477–1491 (2003).

9. West, S. E. H., Schweizer, H. P., Dall, C., Sample, A. K. & Runyen-Janecky, L. J. Construction of improved *Escherichia-Pseudomonas* shuttle vectors derived from pUC18/19 and sequence of the region required for their replication in *Pseudomonas aeruginosa*. *Gene* **148**, 81–86 (1994).

10. Hoang, T. T., Karkhoff-Schweizer, R. R., Kutchma, A. J. & Schweizer, H. P. A broad-host-range Flp-FRT recombination system for site-specific excision of chromosomally-located DNA sequences: application for isolation of unmarked *Pseudomonas aeruginosa* mutants. *Gene* **212**, 77–86 (1998).
